# Genomic insights into influenza A(H1N1)pdm09 virus evolution in Yunnan province, China, during 2018–2023

**DOI:** 10.1186/s12985-026-03238-7

**Published:** 2026-07-03

**Authors:** Meiling Zhang, Xiuping Zhang, Ruize Ni, Yaoyao Chen, Xiaonan Zhao, Jienan Zhou, Yanhong Sun, Xiaoyu Han, Zhaosheng Liu, Chunrui Luo, Xiaoqing Fu, Yong Shao

**Affiliations:** 1 Department of Acute Infectious Diseases Control and Prevention, Yunnan Center for Disease Control and Prevention, Kunming, 650500 China; 2 State Key Laboratory of Genetic Evolution & Animal Models, Kunming Institute of Zoology, Chinese Academy of Sciences, Kunming, 650201 China; 3 Kunming National High-level Biosafety Research Center for Non-Human Primates, Kunming Institute of Zoology, Chinese Academy of Sciences, Kunming, 650107 China

**Keywords:** Yunnan province, Influenza A(H1N1)pdm09 virus, Evolutionary characteristics, Reassortment, Adaptation

## Abstract

Research on regional circulation and evolution of influenza A viruses before and after the COVID-19 pandemic is crucial for informing vaccine updates and antiviral drug development. This study generated 260 new genomic sequences of influenza A(H1N1)pdm09 viruses collected in Yunnan province, China, between 2018 and 2023. Comparative genomics analyses elucidated their evolutionary characteristics and dynamics. Epidemiological analysis identified key risk factors (sex, age, occupation) for influenza infection. Phylogenetic analyses revealed the sequence divergences between the vaccine strains and Yunnan circulating strains, especially in the 2020–2024 influenza seasons. The subclade reassortment events were extremely limited among these sequenced Yunnan strains, suggesting the reassortment may be not a major contributor for the circulation and evolution of influenza A(H1N1)pdm09 viruses in Yunnan during these influenza seasons. We detected the elevated evolutionary pressures acting on the specific gene segments, reflected in increased d_N_/d_S_ ratios, particularly for envelope proteins. Furthermore, numerous amino acid substitutions (e.g., S185I/T) within HA antigenic epitopes and receptor binding sites were identified in most Yunnan strains, indicating potential roles of antigenic drift in modulating viral antigenicity and host adaptation. Notably, 17 amino acid substitutions in HA and NA (including HA: N156K) accumulated to higher frequencies during the 2022–2023 and 2023–2024 seasons. These changes likely represented the molecular signature of contemporary A(H1N1)pdm09 viruses in Yunnan. Collectively, this study explored the molecular evolutionary dynamics of A(H1N1)pdm09 viruses in Yunnan province during diverse influenza seasons, providing new regional data for studying molecular characterization and evolution of A(H1N1)pdm09 within the global surveillance framework.

**Supplementary Information:**

The online version contains supplementary material available at https://doi.org/10.1186/s12985-026-03238-7.

## Introduction

Despite the availability of vaccines and antiviral medications, the World Health Organization (WHO) estimates that seasonal influenza claims 290,000 to 650,000 lives annually [1]. Influenza A viruses are classified into diverse subtypes based on their two surface glycoproteins, hemagglutinin (HA) and neuraminidase (NA). With 18 known HA subtypes and 11 NA subtypes, various combinations generate influenza A viruses exhibiting distinct antigenicity and pathogenicity [2]. Furthermore, reassortment events facilitated by natural hosts (e.g., birds, swine, horses) enable the emergence of novel strains with pandemic potential [3].

Influenza A viruses possess a segmented, negative-sense RNA genome comprising eight segments: HA, NA, matrix protein (M), nucleoprotein (NP), non-structural protein (NS), polymerase acidic (PA), polymerase basic 1 (PB1), and polymerase basic 2 (PB2). These segments encode the viral RNA-dependent RNA polymerase and other proteins regulating viral pathogenicity and host cell apoptosis [2]. The HA protein mediates host cell entry; its antigenic determinants enable receptor binding on host cells, playing crucial roles in viral infection and replication [4]. The NA protein facilitates the release of progeny virions from infected cells and serves as a primary target for antiviral therapies [5]. However, widespread use of neuraminidase inhibitors (NAIs) has driven mutations at key NA amino acid sites, reducing inhibitor affinity and generating drug-resistant strains [6]. The influenza virus polymerase, essential for viral transcription and replication, is also regarded as a highly promising drug target [7]. Continuous viral evolution significantly undermines vaccine efficacy (VE) [8], particularly when antigenic mismatches arise between locally circulating strains and annual vaccine strains. Consequently, the WHO recommends local surveillance to assess seasonal strain patterns, informing the selection of suitable vaccine strains for the Northern and Southern Hemispheres [9]. Therefore, timely evaluation of antigenic divergence between circulating and vaccine strains is essential [10–12].

Yunnan province, situated in southwestern China, shares extensive international borders with Myanmar, Laos, and Vietnam. This strategic location, coupled with its complex geography, may foster distinct regional epidemiological characteristics for influenza viruses. In this study, we conducted comprehensive whole-genome sequencing and comparative genomic analyses of 260 influenza A(H1N1)pdm09 virus strains collected in Yunnan province over diverse monitoring influenza seasons. This research aims to elucidate potential mechanisms and patterns of viral evolution and transmission, and to assess the protective efficacy of influenza vaccines in this region.

## Results

### Demographics of influenza in Yunnan province from 2018 to 2023

**Fig. 1:**
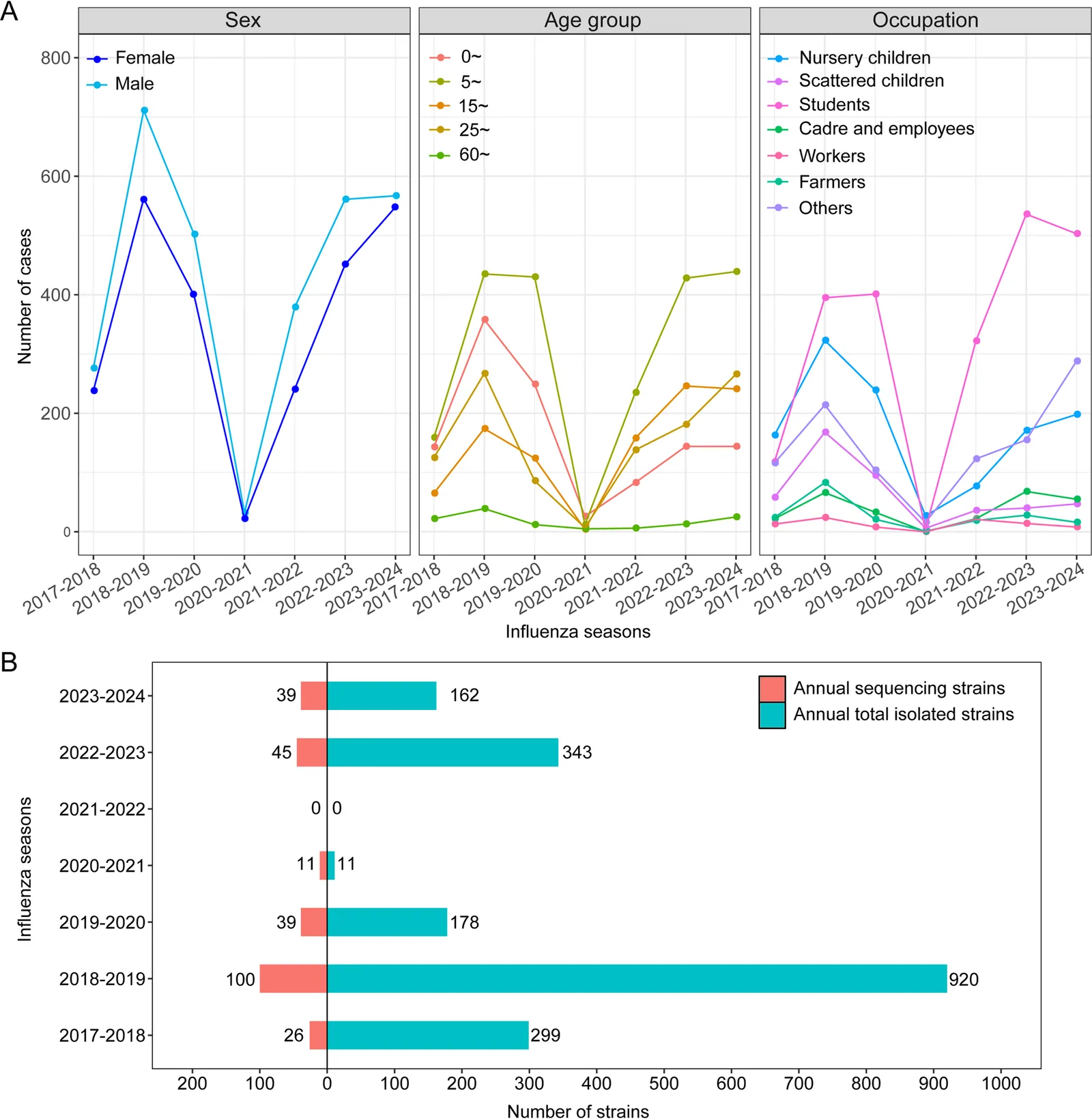
Demographic characteristics of influenza in Yunnan province from seven influenza seasons. (**A**) Demographic distribution of influenza cases by sex, age group, and occupation in Yunnan province from seven influenza seasons. (**B**) Distribution pattern of samples including isolated and sequencing influenza A(H1N1)pdm09 virus strains in Yunnan province from seven influenza seasons. The x-axis represents the number of viral strains and y-axis represents the influenza seasons

This study collected 145,316 specimens from influenza-like illness cases through sentinel hospitals in Yunnan province from 1 January 2018 to 31 December 2023. Totally, 5,489 strains were isolated with 3,906 (2.69%, 3,906/145,316) influenza A virus strains and 1,583 (1.09%, 1,583/145,316) influenza B virus strains. Among 3,906 influenza A virus strains, 1,993 (51.02%, 1,993/3,906) were classified as A(H3N2) and 1,913 (48.98%, 1,913/3,906) were classified as A(H1N1)pdm09 (Table S1). Among the 5,489 influenza cases, the proportion of male patients (55.16%, 3,028/5,489) obviously exceeded that of females (44.84%, 2,461/5,489) (Fig. 1A), with the majority of infections concentrated in the 0 ~ 14 age group, predominantly nursery children and students (Fig. 1A and Table S2). Our results suggest that sex, age and occupation are key factors to be considered in influenza prevention. These findings were consistent with epidemiological studies in other areas or country focusing on influenza A(H3N2) [13] and influenza B [14].

The Yunnan Province covers a total of 16 prefectures/autonomous prefectures. The number of influenza A(H1N1)pdm09 virus strains isolated in different regions varied across different influenza seasons. Therefore, to balance the coverage and representativeness of the virus strain selection, we also considered both temporal and spatial information for the comprehensive sampling in each monitoring influenza season. Taken together, we randomly generated a total of 260 strains from the 1,913 influenza A(H1N1)pdm09 isolates in Yunnan Province from 2018 to 2023 for whole-genome sequencing (Fig. 1B**)**. These sequenced strains represented approximately 25.52% of the total A(H1N1)pdm09 isolates in each monitoring influenza season on average. Notably, influenza circulation was substantially reduced in 2020─2021 (11 strains) and 2021─2022 (no strains) seasons (Fig. 1B), coinciding with COVID-19 mitigation measures including social distancing, mask mandates, and travel restrictions─factors likely contributing to suppressed seasonal influenza transmission [15].

### Phylogenetic analyses and vaccine efficacy estimation of influenza A(H1N1)pdm09 virus strains in Yunnan

**Fig. 2:**
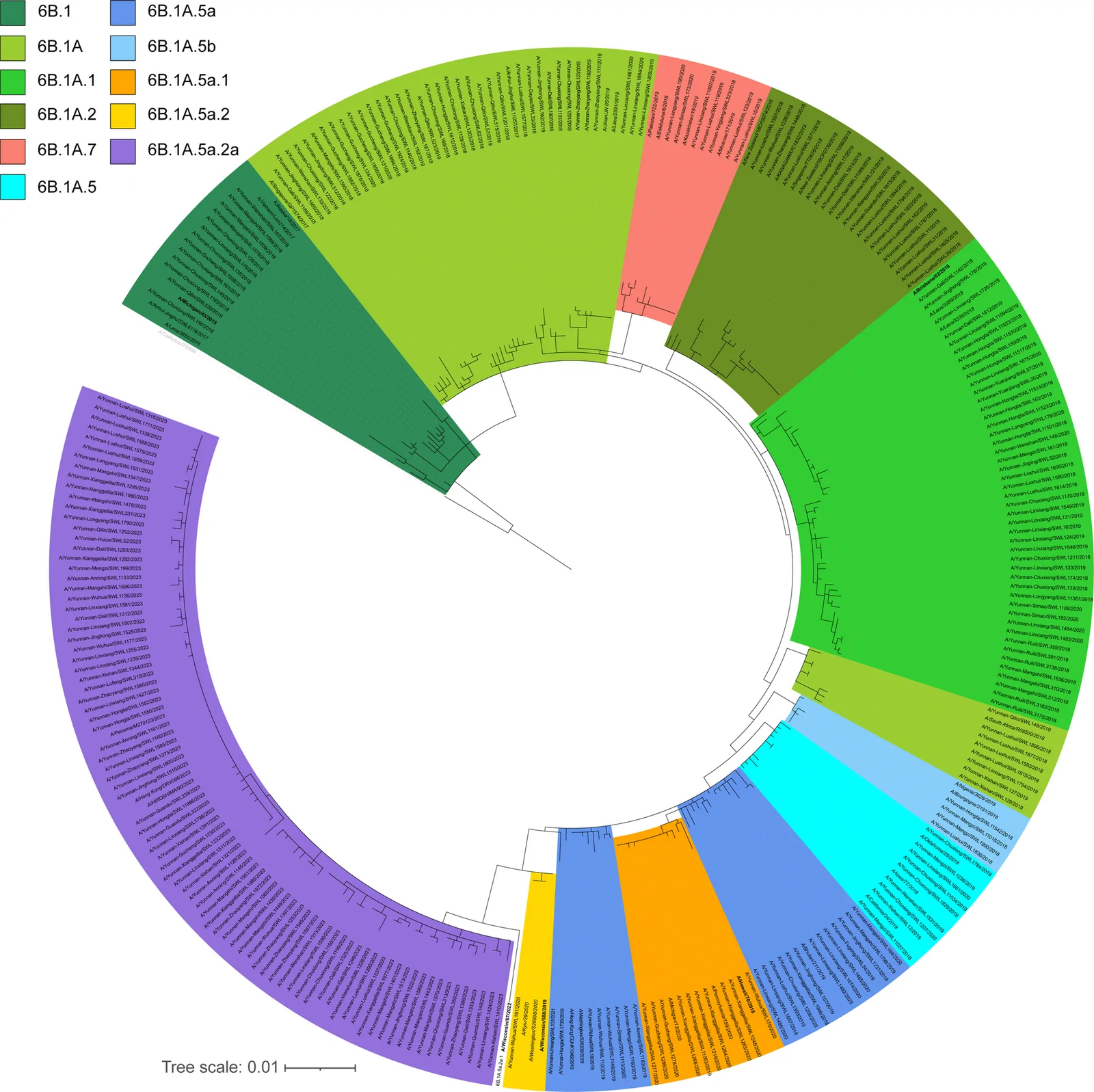
Evolutionary analyses of influenza A(H1N1)pdm09 viruses circulating in Yunnan Province, China, from seven influenza seasons. Maximum-likelihood phylogenetic tree of HA segments of influenza A(H1N1)pdm09 viruses circulating in Yunnan Province, China, from seven influenza seasons. Tree scale refers to the length of the branches in the evolutionary tree. The strains were colored by evolutionary clades including 6B.1, 6B.1 A, 6B.1 A.1, 6B.1 A.2, 6B.1 A.7, 6B.1 A.5, 6B.1 A.5a, 6B.1 A.5b, 6B.1 A.5a.1, 6B.1 A.5a.2, and 6B.1 A.5a.2a. The vaccine strains were marked in bold, and the reference strains were marked in italic. 40 reference strains were utilized to identify these evolutionary clades in this study

Based on the forty reference strains, our HA phylogeny resolved 260 Yunnan sequencing strains into the eleven distinct evolutionary clades/subclades including 6B.1, 6B.1 A, 6B.1 A.1, 6B.1 A.2, 6B.1 A.7, 6B.1 A.5, 6B.1 A.5a, 6B.1 A.5b, 6B.1 A.5a.1, 6B.1 A.5a.2, and 6B.1 A.5a.2a (Fig. 2). Interestingly, some of these clades clearly indicated temporal specificity, suggesting their limited windows of transmission. For example, the clades 6B.1 A.5a.1 and 6B.1 A.5a.2 included Yunnan strains only from the 2019–2020 influenza season. The clade 6B.1 A.5b encompassed Yunnan strains only from the 2018─2019 influenza season (Fig. 2).

Phylogenetic analysis of the HA segment revealed that all 26 circulating Yunnan strains from the 2017–2018 influenza season, were distributed across four evolutionary clades (6B.1, 6B.1 A, 6B.1 A.1, and 6B.1 A.2; Fig. 2). Of the Yunnan isolates collected during the 2017–2018 season, 26.9% (7/26) clustered within clade 6B.1, which encompasses the 2017–2018 vaccine strain A/Michigan/45/2015. Among the Yunnan strains during the 2018–2019 season, they were scattered across eight clades (6B.1, 6B.1 A, 6B.1 A.1, 6B.1 A.2, 6B.1 A.5, 6B.1 A.5a, 6B.1 A.5b, and 6B.1 A.7) and 6% of them (6/100; Fig. 2) belonged to the 6B.1 clade as the 2018─2019 vaccine strain A/Michigan/45/2015. In the 2019─2020 season, the 39 circulating Yunnan strains, exhibiting a highest sequence heterogeneity (Fig. S1), were clustered into eight evolutionary clades (6B.1 A, 6B.1 A.1, 6B.1 A.2, 6B.1 A.5, 6B.1 A.5a, 6B.1 A.5a.1, 6B.1 A.5a.2, and 6B.1 A.7), suggesting greater epidemic complexity of A(H1N1)pdm09 influenza viruses in this season. Further, we found that 23.1% (9/39) of the Yunnan strains during the 2019–2020 season clustered into the 6B.1 A.1 clade together with the vaccine strain A/Brisbane/02/2018. The circulating Yunnan strains in the 2020–2021 season involved four evolutionary clades (6B.1 A, 6B.1 A.1, 6B.1 A.5, and 6B.1 A.5a) and no strain clustered into the evolutionary clade 6B.1 A.5a.1, which covered the vaccine strain A/Hawaii/70/2019 in the 2020–2021 season (Fig. 2). 45 circulating Yunnan strains in the 2022–2023 season and 39 circulating Yunnan strains in the 2023–2024 season together clustered into the clade 6B.1 A.5a.2a, but away from their vaccine strains, A/Wisconsin/588/2019 and A/Wisconsin/67/2022, respectively (Fig. 2). Collectively, these findings highlight the divergence between the vaccine strains and Yunnan circulating strains, especially in the 2020–2024 influenza seasons.

In addition, we further evaluated the vaccine efficacy (VE) for the vaccine strains against the circulating strains in Yunnan from these influenza seasons based on epitopes (A-E) in H1N1, which include 24, 22, 33, 48, and 34 amino acids, respectively (Table 1) [16, 17]. The P_epitope_ method was utilized to estimate the VE of vaccine strains [18]. For Yunnan strains in the 2017–2018 season against the vaccine strain A/Michigan/45/2015, the mean value of P_epitope_ was 0.0349, corresponding to a predicted average VE of 48.85% (Table 1). And this vaccine strain (A/Michigan/45/2015) still maintained a stable average VE of 49% aiming at circulating Yunnan strains in the 2018–2019 season (Table 1). After switching the vaccine strains, during the 2019─2020 and 2020–2021 seasons, the vaccine strains further showed the improved average VE against circulating strains, especially for the vaccine strain A/Hawaii/70/2019 with an average VE of 49.39% (Table 1). Importantly, we noticed that the predicted VE of the 2022─2023 vaccine strain (A/Wisconsin/588/2019) against the 2022─2023 Yunnan circulating strains dropped to 48.04% (Table 1). However, this decreased VE trend had been suppressed by the updated 2023–2024 vaccine strain A/Wisconsin/67/2022, suggesting the necessity of timely update of the influenza vaccine.

**Table 1:** Vaccine efficacy estimation and dominant epitope mutation analysis of influenza A(H1N1)pdm09 strains circulating in Yunnan

Influenza season (*N*)	Vaccine strain	#Strain	Epitope	#Mutation	Residue differences	*P*_epitope_	Vaccine efficacy (%)
2017–2018 (*N* = 26)	A/Michigan/45/2015	1	A	1	S128P/N129D	0.0417	48.04
		8	B	1	S183P	0.0455	47.59
		1	B	1	T185I	0.0455	47.59
		1	C	1	P271Q	0.0303	49.39
		2	C	1	V272A	0.0303	49.39
		19	C	1	I295V	0.0303	49.39
		1	D	1	I96V/I166V	0.0208	50.52
		19	D	1	S164T	0.0208	50.52
		1	D	2	A215G, T216I	0.0417	48.04
		1	E	1	V47I/N84S/I267T	0.0294	49.50
		1	E	2	L70F, A73T	0.0588	46.00
		19	E	1	S74R/K	0.0294	49.50
		2	E	1	A261D	0.0294	49.50
					**Average**	**0.0349**	**48.85**
2018–2019 (*N* = 100)	A/Michigan/45/2015	7	A	1	T137A	0.0417	48.04
		14	A	1	H143Y	0.0417	48.04
		15	A	1	N146D	0.0417	48.04
		4	A	1	E252D	0.0417	48.04
		1	B	1	S124L/V190I	0.0455	47.59
		2	B	1	P154L/H155Y/A156T	0.0455	47.59
		9	B	1	E189K	0.0455	47.59
		8	B	1	G195A	0.0455	47.59
		1	C	1	R269K/M274L/P288L	0.0303	49.39
		17	C	1	T273I	0.0303	49.39
		2	C	1	R276K	0.0303	49.39
		19	C	1	N277D	0.0303	49.39
		4	C	1	A278D/A278V/I303L	0.0303	49.39
		3	D	1	I113V/S207G/S207R /S207I	0.0208	50.52
		1	D	1	I166V/S174L/A212T /R222G/E241K	0.0208	50.52
		59	D	1	S200P	0.0208	50.52
		25	D	1	T202I	0.0208	50.52
		1	E	1	K57N/S86P	0.0294	49.50
		2	E	1	N73S	0.0294	49.50
		4	E	1	I257V	0.0294	49.50
					**Average**	**0.0336**	**49.00**
2019–2020 (*N* = 39)	A/Brisbane/02/2018	2	A	1	T137A	0.0417	48.04
		3	A	1	H143Y	0.0417	48.04
		20	A	1	N146D	0.0417	48.04
		1	A	1	K147N	0.0417	48.04
		5	C	1	T273I	0.0303	49.39
		18	C	1	N277D	0.0303	49.39
		9	C	1	P288S	0.0303	49.39
		2	C	1	I303L	0.0303	49.39
		2	D	1	I113V	0.0208	50.52
		1	D	2	N173K, K226M	0.0417	48.04
		7	D	1	P200S	0.0208	50.52
		24	D	1	T202I	0.0208	50.52
		10	D	2	D204A, Q206E	0.0417	48.04
		2	E	1	I257V	0.0294	49.50
		1	E	1	V267A	0.0294	49.50
					**Average**	**0.0328**	**49.09**
2020–2021 (*N* = 11)	A/Hawaii/70/2019	5	A	1	D146N	0.0417	48.04
		1	B	1	V190I	0.0455	47.59
		2	C	1	T273I	0.0303	49.39
		5	C	1	D277N	0.0303	49.39
		1	D	1	N173K	0.0208	50.52
		2	D	1	P200S	0.0208	50.52
		5	D	1	I202T	0.0208	50.52
		11	D	2	A204D, E206Q	0.0417	48.04
		1	D	1	A212E	0.0208	50.52
					**Average**	**0.0303**	**49.39**
2022–2023 (*N* = 45)	A/Wisconsin/588/2019	2	B	1	A156D	0.0455	47.59
		1	B	1	V190I	0.0455	47.59
		45	C	1	R276K	0.0303	49.39
		1	D	1	T89K/V237L	0.0208	50.52
		45	D	2	Q206E, E241A/S	0.0417	48.04
		45	E	1	K71Q	0.0294	49.50
					**Average**	**0.0355**	**48.77**
2023–2024 (*N* = 39)	A/Wisconsin/67/2022	38	B	1	S154P	0.0455	47.59
		1	B	1	A156D/E189K	0.0455	47.59
		39	C	1	E277D	0.0303	49.39
		1	D	1	V169I	0.0208	50.52
		4	D	1	A241S	0.0208	50.52
		1	E	1	Q71H	0.0294	49.50
					**Average**	**0.0320**	**49.19**

These influenza seasons covered the samples from 1 January 2018 to 31 December 2023

### Potential limited subclade reassortment events occurred among the circulating Yunnan virus strains

Phylogenies of the seven remaining segments (M, NA, NP, NS, PA, PB1, and PB2) for the circulating Yunnan strains during six influenza seasons were reconstructed using maximum likelihood. The topologies of all eight segment-based phylogenies demonstrated broadly consistent evolutionary patterns (Figs. 2 and S2).

**Fig. 3:**
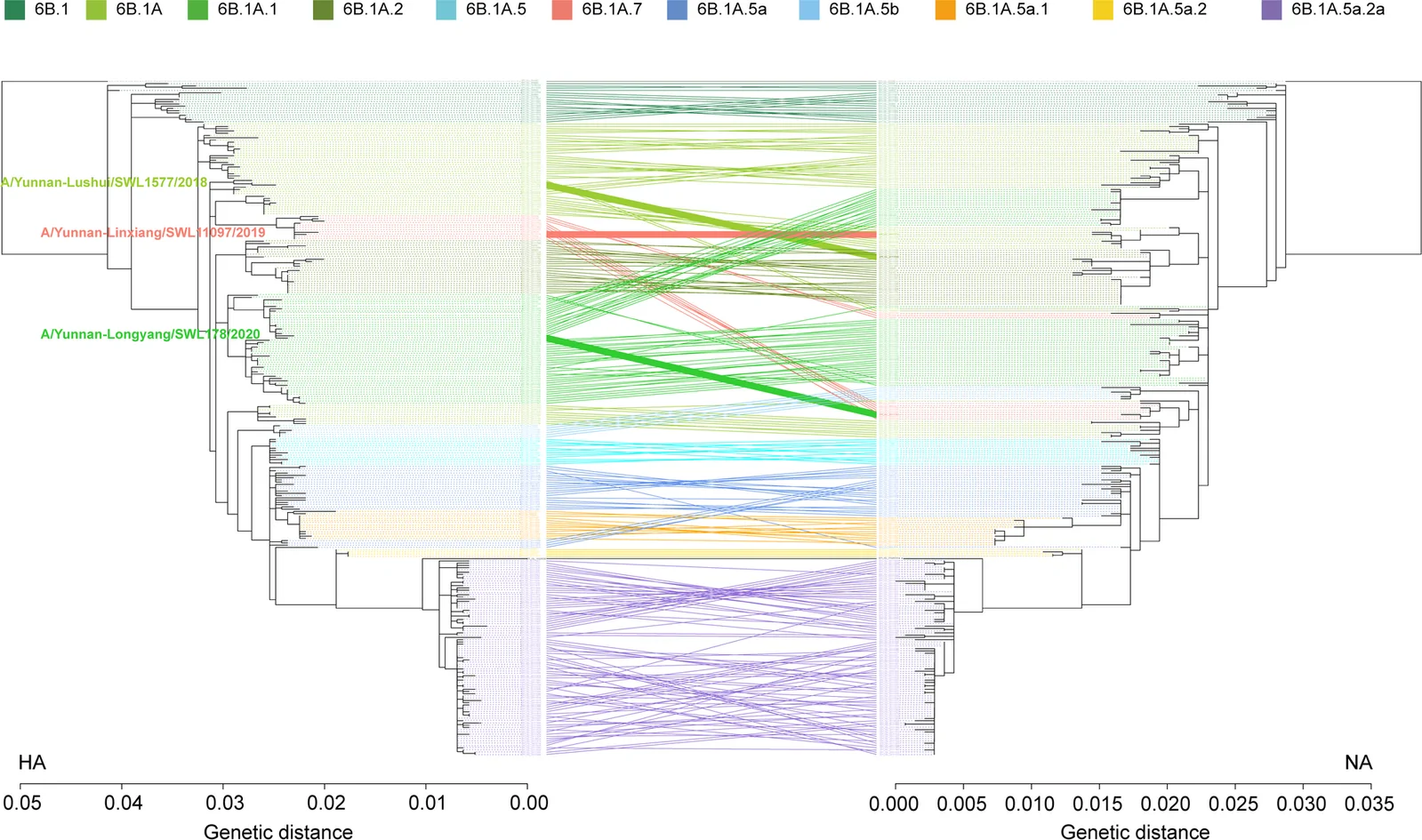
Reassortment events between HA and NA segments of influenza A(H1N1)pdm09 viruses circulating in Yunnan province, China, from 2018 to 2023. Tanglegram was displayed with the ML HA tree on the left side and the mirrored tree of NA on the right side. Twines between both trees were colored according to the evolutionary clades. GiRaF and phylogeny method were used to detect reassortment events between segments. The three reassortment strains (A/Yunnan-Lushui/SWL1577/2018, A/Yunnan-Linxiang/SWL11097/2019, and A/Yunnan-Longyang/SWL178/2020) were highlighted and further connected by the thick line between HA and NA phylogeny topologies

By integrating the Graph Incompatibility-based Reassortment Finder (GiRaF) [19] and the IQ-Tree [20] phylogeny analyses across eight segments, we observed that most viruses remained stable within their original evolutionary clades (6B.1, 6B.1 A, 6B.1 A.1, 6B.1 A.2, 6B.1 A.7, 6B.1 A.5, 6B.1 A.5a, 6B.1 A.5b, 6B.1 A.5a.1, 6B.1 A.5a.2, and 6B.1 A.5a.2a), though occasional limited positional shifts between these evolutionary clades or subclades (Figs. 3 and S2). Deeply, the viruses of seven clades (6B.1, 6B.1 A.5b, 6B.1 A.5, 6B.1 A.5a, 6B.1 A.5a.1, 6B.1 A.5a.2, and 6B.1 A.5a.2a) were generally stable across all eight segment phylogenies (Figs. 3 and S2), indicating no reassortment event occurred. Further, we found the potential reassortments mainly occurred in other four clades including 6B.1 A, 6B.1 A.2, 6B.1 A.7, and 6B.1 A.1. For example, our analyses revealed five potential reassortment strains with distinct segment constellations supported by integrating two strategies including phylogeny (topology structures or bootstrap values) and GiRaF (Conf > 0.9) (See Methods, Figs. 3 and S2-S7). A/Yunnan-Lushui/SWL1577/2018 showed incongruent topologies between its HA/M segments (in clade 6B.1 A) and the NA/NP/PA/PB1/PB2 segments (in clade 6B.1 A.2) (Figs. 3 and S2-S7). Similarly, A/Yunnan-Linxiang/SWL11097/2019 exhibited discordant placements, clustering its HA/NP/NS/PB2 segments in the clade 6B.1 A.7, while its NA/M/PA/PB1 segments fell within the clade 6B.1 A (Figs. 3 and S2-S7). For A/Yunnan-Longyang/SWL178/2020, the HA/NP/PB1/PB2 segments grouped with clade 6B.1 A.1, whereas the NA/M/NS/PA segments were placed in clade the 6B.1 A.7 (Figs. 3 and S2-S7). A/Yunnan-Qilin/SWL148/2019 had incongruent topologies between M (in 6B.1 A.7) and other segments (HA, NA, NP, NS, PA, PB1, and PB2; in 6B.1 A) (Figs. S2-S7). Finally, A/Yunnan-Longyang/SWL190/2020 showed discordant placements in phylogenies between PA (in 6B.1 A) and other segments (HA, NA, M, NP, NS, PB1 and PB2; in 6B.1 A.7) (Figs. S2-S7).

Furthermore, three additional Yunnan strains—A/Yunnan-Lushui/SWL1871/2018, A/Yunnan-Wenshan/SWL148/2020, and A/Yunnan-Dali/SWL1142/2018—exhibited potential reassortment signals solely based on phylogenetic topology analyses (Table S3), albeit without support from the GiRaF algorithm. Additionally, we identified putative reassortment events in seven reference strains and one vaccine strain (Table S3); these were corroborated by both analytical approaches, although some were uniquely detected via phylogenetic inference.

In summary, only 3–8 of the 260 sequenced strains exhibited detectable reassortment events. The relative scarcity of such events indicates that reassortment was not a major driver of A(H1N1)pdm09 evolution in Yunnan during the study period.

### Adaptive evolution of influenza A(H1N1)pdm09 Yunnan virus strains

**Fig. 4:**
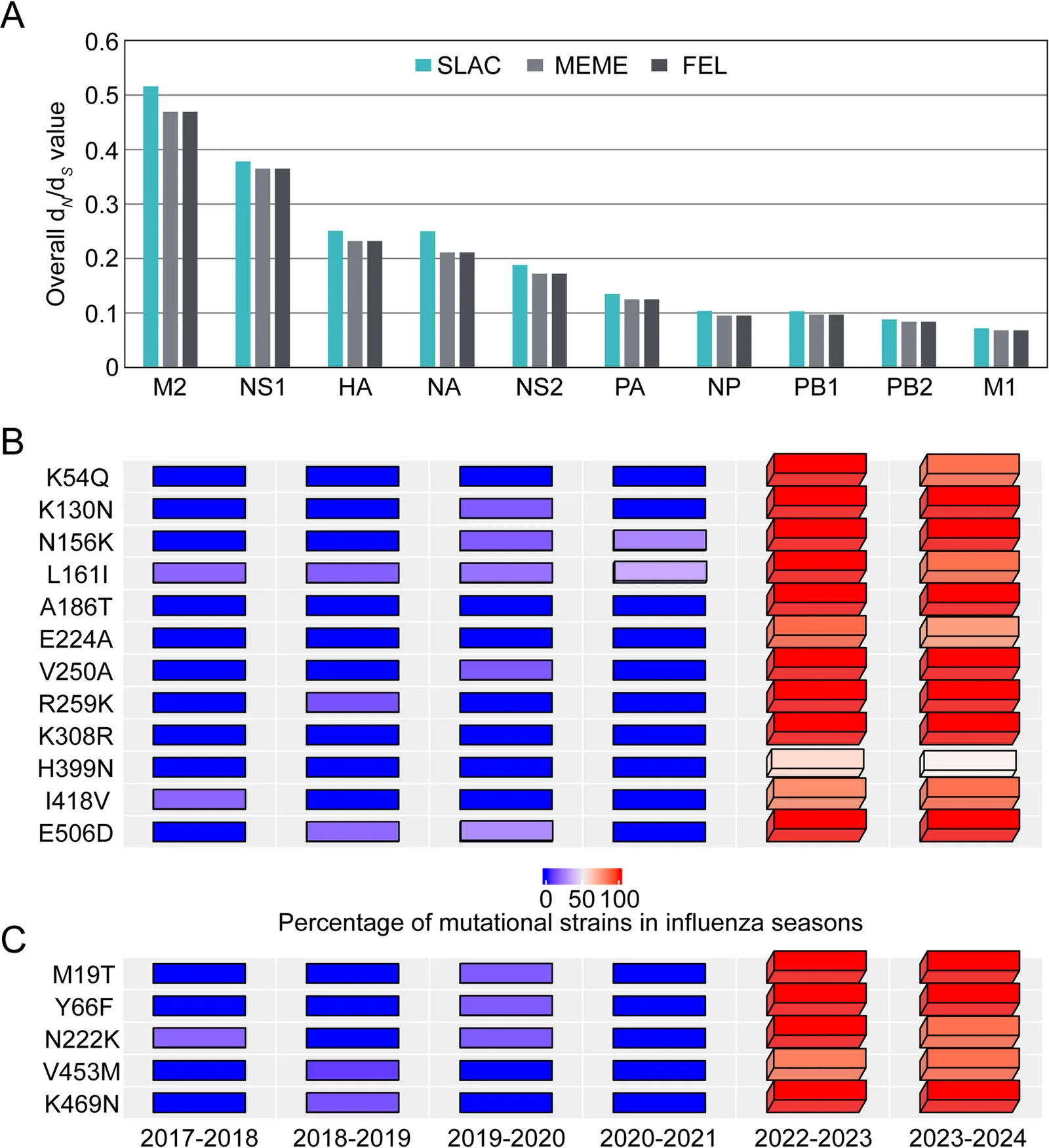
Evolutionary pressure and mutation of Influenza A(H1N1)pdm09 Yunnan virus strains. (**A**) The overall d_N_/d_S_ values of different gene segments for Influenza A(H1N1)pdm09 Yunnan virus strains based on three algorithms including SLAC, MEME, and FEL. (**B**) Distributional percentage of 12 amino acid substitutions on HA for the circulating influenza A(H1N1)pdm09 Yunnan strains across six influenza seasons. (**C**) Distributional percentage of 5 amino acid substitutions on NA for the circulating influenza A(H1N1)pdm09 Yunnan strains across six influenza seasons

Host-pathogen interactions drive pathogen adaptive evolution [21]. Consistent with evolutionary constraints observed in H3N2 [22, 23], we found that HA and NA together with M2 and NS1 in influenza A(H1N1)pdm09 Yunnan virus strains harbored the elevated selective pressures with d_N_/d_S_ > 0.2 than those of other viral segments (Fig. 4A and Table S4). Notably, the increased antibody accessibility of envelope proteins (HA, NA, and M2) likely explains their higher d_N_/d_S_ values relative to internal proteins [22].

Facing intense host-derived selective pressures, influenza A(H1N1)pdm09 pathogens accumulate mutations to maintain fitness while evading innate and adaptive immunity. Analysis of Yunnan strains from six influenza seasons (2017–2018, 2018–2019, 2019–2020, 2020–2021, 2022–2023, and 2023–2024) revealed multiple amino acid substitutions across segments, with HA proteins exhibiting 18 recurrent mutations (S74R, P83S, S84N, D97N, S162N, K163Q, S164T, S183P, S185I/T, S203T, I216T, A256T, K283E, I295V, I321V, E374K, S451N, E499K) in > 73% of strains compared to A/California/7/2009 (Table S5). Among these amino acid substitutions, 11 (61%) were located within 4 antigenic epitopes (epitope B: S162N, S183P, and S185I/T; epitope C: K283E and I295V; epitope D: K163Q, S164T, and I216T; epitope E: S74R, P83S and S84N). The mutation, S185I/T, was also located in the receptor binding sites (RBSs) (positions 184–191, 218–225, 131–135, Y91, W150, H180 and Y192) [24]. Crucially, 12 amino acid substitutions (K54Q, K130N, N156K, L161I, A186T, E224A, V250A, R259K, K308R, H399N, I418V, E506D; Fig. 4B) possessed a higher percentage in the Yunnan strains during the 2022–2023 and 2023–2024 seasons compared with those of other influenza seasons, and half of 12 substitutions resided in epitopes (K54Q, N156K, A186T, E224A, R259K, and K308R)─with A186T/E224A also in RBSs. Importantly, five epitope mutations (K54Q, A186T, E224A, R259K, and K308R) were concurrently reported in Sri Lanka from 2023 [25]; such epitope alterations likely drive antigenic drift and compromise vaccine-induced immunity [26, 27].

The NA protein facilitates viral particle release from host cells, serving as a critical target for antivirals and antibodies [5]. Relative to A/California/7/2009, NA proteins of A(H1N1)pdm09 strains circulating in Yunnan over six influenza seasons accumulated 107 distinct mutations (≥ 1 isolate each; Table S6). All Yunnan strains harbored the N248D mutation, known to enhance low-pH stability in A(H1N1)pdm09 viruses [28]. Double mutations V241I and N369K occurred in all Yunnan strains, and they have been involved in enhancing the in vitro activity and adaptability of A(H1N1)pdm09 virus in ferrets [29]. The mutations (N248D, N369K and N449D) also affect the NA surface to lose its binding to human monoclonal antibodies, resulting in viral immune escape [30]. Interestingly, we identified that five amino acid substitutions harbored a higher percentage in Yunnan strains during the 2022–2023 and 2023–2024 influenza seasons compared with those of other seasons (M19T, Y66F, N222K, V453M, and K469N; Fig. 4C). Studies have demonstrated that mutations in the NA protein—including S110F, I117R, E119A/D/G/V, Q136K/R, R152K, D199E/G/Y, I223K/L/M/R/T, S247G/R, H275Y, R293K, N295S, I427T, I436N, P458T, I223V, S247N, as well as the combinations G147R+H275Y and Q313R+I427T—can confer reduced susceptibility to neuraminidase inhibitors such as oseltamivir, zanamivir, peramivir, and laninamivir [31]. Only A/Yunnan-Mangshi/SWL1609/2018 in the 2018─2019 influenza season carried the S247N resistance mutation without concurrent I233V (Table S6). Mutations at key positions included E119K in three strains (one in the 2018─2019 season and two in the 2023─2024 season), I223M in two strains during the 2018–2019 season, I117M in 15 strains (11 strains in the 2018–2019 season, 3 strains in the 2019–2020 season, one in the 2020-2021season), and I436V in 20 strains (one in the 2017–2018 season, 16 strains in the 2018–2019 season, 3 strains in the 2019–2020 season), and these mutations deserved further functional studies.

Additionally, we characterized mutations across six proteins (M2, NS1, NP, PA, PB1, and PB2) in A(H1N1)pdm09 Yunnan strains (Table S7). All Yunnan strains carried M2-S31N conferring amantadine resistance [32], with ten strains exhibiting adamantane-resistant V27A [32]. Universal NS1-I123V/L mutations within its functional domain enhance adaptation and virulence [33]. A previous study has indicated that positions 313 and 357 may play crucial roles in the mitochondrial autophagy mediated by the Influenza A virus nucleoproteins (NP) [34]. In this study, the mutation V313I was observed in a small subset of Yunnan virus strains and the novel K357R mutation was identified in one 2018─2019 Yunnan strain. Thus, the deep investigation could determine their potential impacts on viral replication and pathogenicity. The proteins (PA, PB1, and PB2) form a polymerase complex that utilizes the cap-snatching mechanism to facilitate the replication and transcription of viral RNA in the nucleus of infected cells [35]. None of the mutations known to confer resistance to baloxavir marboxil—specifically E23G/K/R, K34R, A37T, I38F/L/M/S/T/V, E198K, and E199D/G—were detected in any of the Yunnan A(H1N1)pdm09 strains isolated in these monitoring influenza seasons [36]. All Yunnan strains possessed the PA-P224S mutation, which may improve viral virulence by affecting the transport and accumulation of PA proteins in the nucleus of virus-infected cells [37]. All the PB1 open reading frames of the Yunnan strains in these influenza seasons contained a stop codon at position 12, as previously found in other studies, expressing the truncated PB1-F2 protein (11aa), which has minimal impact on viral pathogenicity in animal models [38, 39]. The mutation T588I on PB2 of A(H1N1)pdm09 virus was commonly recognized as a virulence marker [40]. The single 2022─2023 strain in this study carried this mutation on the PB2 protein, which may exacerbate clinical symptoms in infected individuals. Existing data have shown that the mutations, E627K and D701N, on PB2 protein contribute to enhancing viral adaptation and pathogenesis to mammals [4, 41]. In this study, one 2018─2019 strain and one 2017─2018 strain harbored two novel mutations (E627V and D701E) in its PB2 protein, respectively and their roles require further functional characterization.

## Discussion

Influenza virus remains a major public health threat, capable of triggering seasonal epidemics and unpredictable pandemics [42]. Our epidemiological analysis of Yunnan Province (2017─2024) revealed significant sex-based disparities, with males exhibiting higher infection rates than females─highlighting the impact of biological sex on influenza virus pathogenesis [43]. We further identified the 0–14 age bracket (particularly nursery children and students) as the demographic with highest infection rates, aligning with prior reports [44]. These findings underscore the critical need to prioritize vaccination strategies for these vulnerable populations to mitigate disease burden.

By tracing A(H1N1)pdm09 evolution in Yunnan pre- and post-COVID-19, we revealed the levels of sequence divergences between the vaccine strains and Yunnan circulating strains. Our findings support genomic surveillance as a vital tool for assessing vaccine effectiveness so that the potential candidate vaccine strains with better antigenic match to the circulating strains can be selected and adjusted in a timely manner. As a critical evolutionary driver, genetic reassortment across influenza Intra- and inter-subtype enables viral adaptation [45] and reassortment events among multiple lineages may enhance transmissibility and the pandemic potential [46]. In this study, we newly generated the whole-genome information of 260 influenza A(H1N1)pdm09 virus strains in Yunnan during diverse influenza seasons and performed a series of comparative genomics analyses. And the potential subclade reassortment events were detected in A(H1N1)pdm09 strains from Yunnan Province. Such events were infrequent in Yunnan strains, representing only 1.9─3.1% (5–8/260) of all sequenced Yunnan isolates [47]. Therefore, we inferred the limited reassortment events may contribute to few transmission complexity during this period.

Host-pathogen interactions exert continuous selective pressure favoring viral adaptation [48]. The higher d_N_/d_S_ ratios for the segments coding envelope proteins (e.g., HA, NA, M2) of A(H1N1)pdm09 Yunnan strains were found in this study and these may involve the selective pressures from host antibodies [22]. Among 18 amino acid substitutions located in the most of the Yunnan strains, 11 amino acid mutations (> 61%) were found in the antigen epitopes regions of the HA protein, which may impact the antigenicity of the prevailing influenza A(H1N1)pdm09 strains in Yunnan. The substitution (S185I/T) occupy both antigenic and receptor binding sites, suggesting that it may allow the virus to improve its antigenic structure or adaptability [26, 49, 50]. The amino acid substitutions in the NA protein play a crucial role in the development of resistance to neuraminidase inhibitors [31]. We have identified some amino acid substitutions at key sites (e.g., 119, 247) related to neuraminidase inhibitor resistance in some of the Yunnan virus strains. Whether these mutations will have an impact on the virus resistance is worthy of our continued further research. Importantly, we identified some amino acid substitutions on HA and NA segments, which harbored a high frequency in A(H1N1)pdm09 Yunnan strains from the 2022–2023 and 2023–2024 influenza seasons, suggesting these may represent their crucial characteristics of viral mutation and evolution (e.g., N156K in HA). Overall, all Yunnan strains remained resistant to adamantanes but retained sensitivity to neuraminidase inhibitors and baloxavir marboxil, as in previous studies [32, 51–54].

In conclusion, through large-scale comparative genomics analysis, this study revealed the evolutionary complexity dynamics, rare subclade reassortment events, and adaptations for the A(H1N1)pdm09 Yunnan strains from diverse influenza seasons and further provides insights into histories of A(H1N1)pdm09 evolution in Yunnan, China, which will aid for vaccine updates and antiviral treatment strategies.

## Methods and materials

### Specimen collection and virus isolation

Surveillance was conducted on patients with influenza-like illness (ILI)─defined by sudden high fever (≥ 38℃) and respiratory symptoms (e.g., cough, sore throat)─admitted to sentinel hospitals in Yunnan Province, China, from January 1, 2018, to December 31, 2023. Throat swab samples were collected with informed consent from patients or guardians, stored in viral transport medium, and transported within 24 hours to Yunnan provincial influenza surveillance network laboratories. RNA extraction was performed using an automated nucleic acid extraction system (Tianlong NP968, Xian, China), followed by real-time RT-PCR for influenza virus detection using specific primers and probes: FluA-Forward (5’-GACCRATCCTGTCACCTCTGAC-3’), FluA-Reverse (5’-GGGCATTYTGGACAAAKCGTCTACG-3’), FluA-Probe (5’-TGCAGTCCTCGCTCACTGGGCACG-3’), H1-Forward (5’-GGGTAGCCCCATTGCAT-3’), H1-Reverse (5’-AGAGTGATTCACACTCTGGATTTC-3’), and H1-Probe (5’-TGGGTAAATGTAACATTGCTGGCTGG-3’). Positive samples (1–2 mL) were inoculated into Madin-Darby Canine Kidney (MDCK) cells (Chinese National Influenza Center origin) in T25 flasks (Corning) and incubated at 35 ℃/5% CO_2_ for 1–2 h; after PBS wash (Gibco, 20012050), cells were maintained in DMEM (Gibco, 11965118) supplemented with penicillin (100 U/mL), streptomycin (100 µg/mL), and TPCK-trypsin (2 µg/mL). Cytopathic effect was monitored for 4–7 days; harvested viruses were confirmed by hemagglutination assay involving serial twofold dilution in PBS (50 µL/well, duplicate), addition of 50 µL 1% guinea pig erythrocytes in PBS, and room temperature incubation (30–45 min). Subtyping used hemagglutination inhibition tests with ferret antisera against Northern Hemisphere vaccine strains (2017─2024, Chinese National Influenza Center, Beijing). All procedures followed the National Influenza Surveillance Technical Guidelines (2017 Edition); results underwent peer review by the Yunnan Center for Disease Control and Prevention prior to final confirmation by the Chinese National Influenza Center.

### Whole-genome sequencing of influenza A(H1N1)pdm09 viruses

Nucleic acids from isolated strains were extracted using an automated nucleic acid extractor (BioPerfectus SAW-96, Jiangsu, China). cDNA synthesis and PCR amplification utilized the SuperScript™ III One-Step RT-PCR System with Platinum™ Taq High Fidelity (Invitrogen, Carlsbad, CA, USA, 12574035) and amplification primers Uni-12/Inf1 (A: 5’-GGGGGGAGCAAAAGCAGG-3’), Uni-12/Inf3 (B: 5’-GGGGGGAGCGAAAGCAGG-3’), and Uni-13/Inf1 (C: 5’-CGGGTTATTAGTAGAAACAAGG-3’). Reaction setup and cycling conditions followed the National Influenza Surveillance Technical Guidelines (2017 Edition). Amplification products underwent purification with magnetic beads (Beckman Coulter, A63881), quantification via Qubit™ dsDNA HS Assay Kit (Invitrogen, Q32854), and dilution to 0.2 ng/µL. Sequencing libraries were prepared with the Nextera XT DNA Library Prep Kit (Illumina, FC-131-1096) according to manufacturer instructions. Libraries were pooled, denatured, diluted to 1.1 pM, and loaded into MiniSeq High Output reagent cartridges (300 cycles; FC-420-1003). Sequencing proceeded on the Illumina MiniSeq platform using the system guide. Raw paired-end reads were processed in CLC Genomics Workbench 23 (Qiagen, Aarhus, Denmark) with quality trimming (Q ≥ 0.05; ambiguous nucleotides ≤ 2) and adapter removal (5’ 5 nt; 3’ 18 nt). High-quality reads were mapped to vaccine strains from corresponding years using parameters: match score = 1, mismatch cost = 2 (linear gap cost), length fraction = 0.5, similarity fraction = 0.8, auto-detected paired distance, and non-specific matches handling: mapped randomly. Consensus sequences achieving ≥ 3000X sequencing depth were generated for downstream analysis.

### Phylogenetic analysis of influenza A(H1N1)pdm09 viruses

Forty global reference strains─including five WHO-recommended Northern Hemisphere vaccine strains (2017─2024) [A/Michigan/45/2015 (H1N1)pdm09-like virus (2017─2018), A/Michigan/45/2015 (H1N1)pdm09-like virus (2018─2019), A/Brisbane/02/2018 (H1N1)pdm09-like virus (2019─2020), A/Hawaii/70/2019 (H1N1)pdm09-like virus (2020─2021), A/Wisconsin/588/2019 (H1N1)pdm09-like virus (2021─2022), A/Wisconsin/588/2019 (H1N1)pdm09-like virus (2022─2023), and A/Wisconsin/67/2022 (H1N1)pdm09-like virus (2023─2024)]─were retrieved from GISAID (https://gisaid.org/) and incorporated into our phylogenetic analysis. Multiple sequence alignments were generated using MAFFT v7 with default parameters [55]. Phylogenetic trees for all eight gene segments (HA, NA, M, NP, NS, PA, PB1, and PB2) were reconstructed via maximum likelihood in IQ-Tree v2.3.6 [20]. The algorithm initiated the tree reconstruction after assessment and selection of the optimal nucleotide substitution model. Topological robustness was assessed with 1,000 nonparametric bootstrap replicates. Consensus trees were visualized using iTOL v7 (https://itol.embl.de/) [56]. Clade assignments were based on the NextClade software (https://clades.nextstrain.org/).

### Reassortment detection of influenza A(H1N1)pdm09 viruses

Reassortment events were identified by the GiRaF [19] algorithm using the default parameters. The reassortment confidence level was further controlled by a cutoff of > 0.9. Further, A library “dendextend” of R (https://talgalili.github.io/dendextend/) was utilized to generate tanglegrams by using the phylogenetic ML trees constructed by the IQ-Tree v2.3.6 [20]. The phylogeny topology and bootstrap value also were used to validate the reassortment events.

### Nucleotide homologous and amino acid substitution analysis of viral genomic sequences

Nucleotide divergence of influenza A(H1N1)pdm09 viruses was determined using BLAST [57] through pairwise identity comparisons of circulating strains. This study focused on homologous analyses of HA and NA segments in Yunnan A(H1N1)pdm09 viruses. Amino acid substitutions across all eight gene segments were analyzed in MEGA X [58], relative to the A/California/7/2009 reference strain. Based on homologous sequence analyses, mutations in H1 epitopes (Sa, Sb, Ca1, Ca2, Cb) were mapped to corresponding H3 epitopes (B + D, B, C + D, A + D, and E) as previously defined [12, 16, 59].

### Evolutionary pressure analyses of influenza A(H1N1)pdm09 viruses

To evaluate codons under selective constraint during viral adaptation, we calculated the non-synonymous to synonymous substitution rate ratio (d_N_/d_S_) in this study. Selection pressures acting on all gene segments of influenza A(H1N1)pdm09 viruses were assessed using three methods in HYPHY [60]: Fixed Effects Likelihood (FEL), Single-Likelihood Ancestor Counting (SLAC), and Fast Unconstrained Bayesian Approximation (FUBAR). These analyses were conducted through the Datamonkey [61] webserver (https://www.datamonkey.org/).

### Prediction of vaccine efficacy

The P_epitope_ method estimates influenza VE by calculating the proportion of amino acid substitutions occurring within HA epitopes relative to their total length [18]. To determine antigenic distance between circulating and vaccine strains, we utilized five A(H1N1)pdm09 epitopes (A-E) mapped to corresponding A/H3N2 epitopes [16]. VE for influenza A(H1N1)pdm09 was calculated as: VE = (0.53–1.19 × P_epitope_) × 100 [17, 59].

## Supplementary Information

Below is the link to the electronic supplementary material.

Supplementary Material 1.

Supplementary Material 2.

Supplementary Material 3.

Supplementary Material 4.

Supplementary Material 5.

Supplementary Material 6.

Supplementary Material 7.

Supplementary Material 8.

Supplementary Material 9.

Supplementary Material 10.

Supplementary Material 11.

## Data Availability

The genome sequencing data from 260 influenza A(H1N1)pdm09 virus strains in Yunnan from 2018 to 2023 have been deposited in Table S8 with the accession numbers, and they are available in GISAID (https://gisaid.org/).
