# Supplementary material for: Genomic insights into influenza A(H1N1)pdm09 virus evolution in Yunnan province, China, during 2018–2023
Source: Virol J. 2026 Jul 3;23:222. doi: 10.1186/s12985-026-03238-7 (PMC13602790; doi:10.1186/s12985-026-03238-7)
Supplement: Supplementary file 1 [file 12985_2026_3238_MOESM1_ESM.pdf]

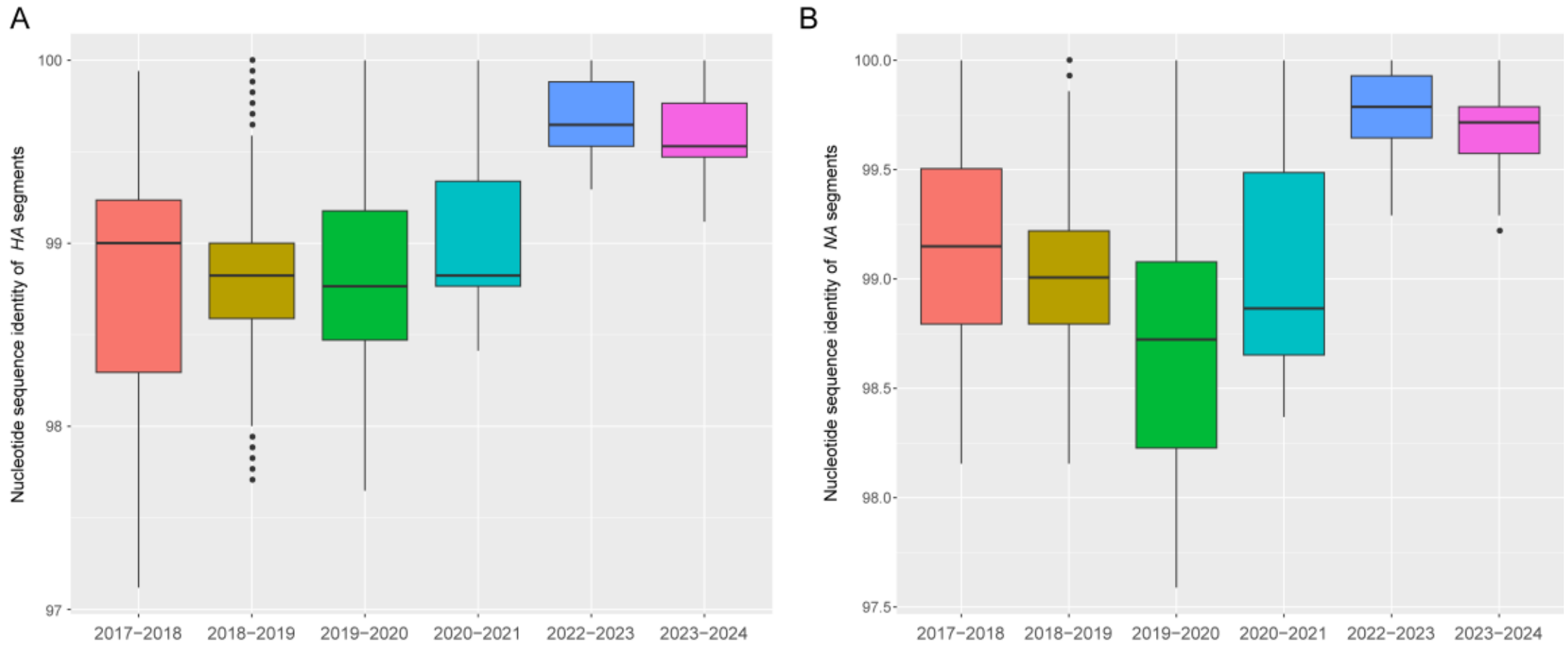

Figure S1. Sequence homology analyses of HA and NA segments of influenza A(H1N1)pdm09 viruses in Yunnan Province, China. (A) Nucleotide sequence identity of *HA* segments from six influenza seasons. (B) Nucleotide sequence identity of *NA* segments from six influenza seasons. Due to extremely limited influenza A(H1N1)pdm09 virus strains circulating in Yunnan Province, China, during 2021-2022, they were not shown here.

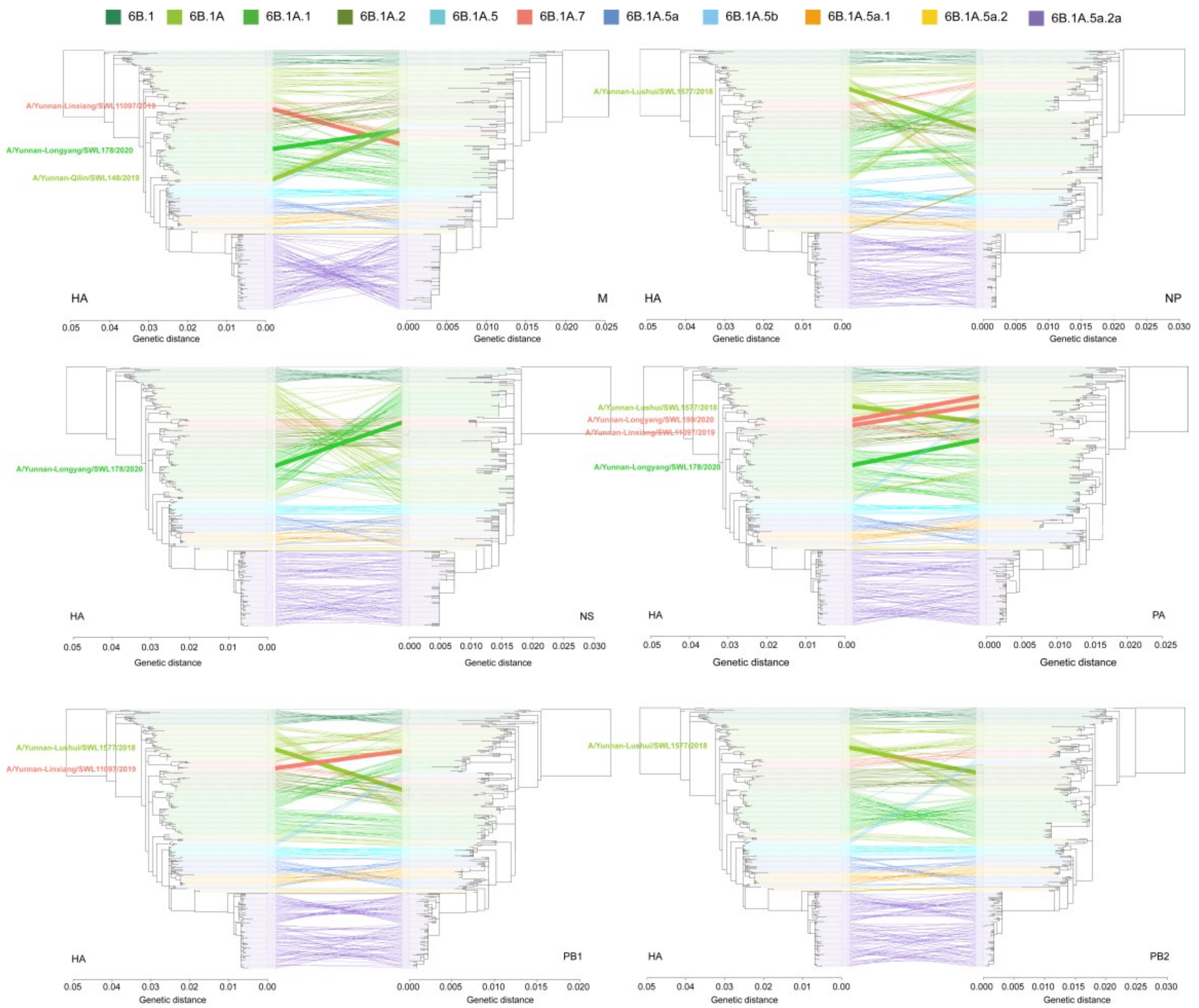

Figure S2. Potential reassortment events between HA and other segments of influenza A(H1N1)pdm09 viruses circulating in Yunnan Province, China, from different influenza seasons. Tanglegrams were displayed with the ML HA tree on the left side and the mirrored trees of M, NP, NS, PA, PB1 and PB2 on the right side. GiRaF and phylogeny were used to detect reassortment events between segments. The five Yunnan reassortment strains (A/Yunnan-Lushui/SWL1577/2018, A/Yunnan-Linxiang/SWL11097/2019, A/Yunnan-Qilin/SWL148/2019, A/Yunnan-Longyang/SWL190/2020, and A/Yunnan-Longyang/SWL178/2020) supported by phylogeny and GiRaF (Conf > 0.9) were highlighted and further connected by the thick line between HA and other phylogeny topologies. The genetic distance was showed in each ML phylogeny.

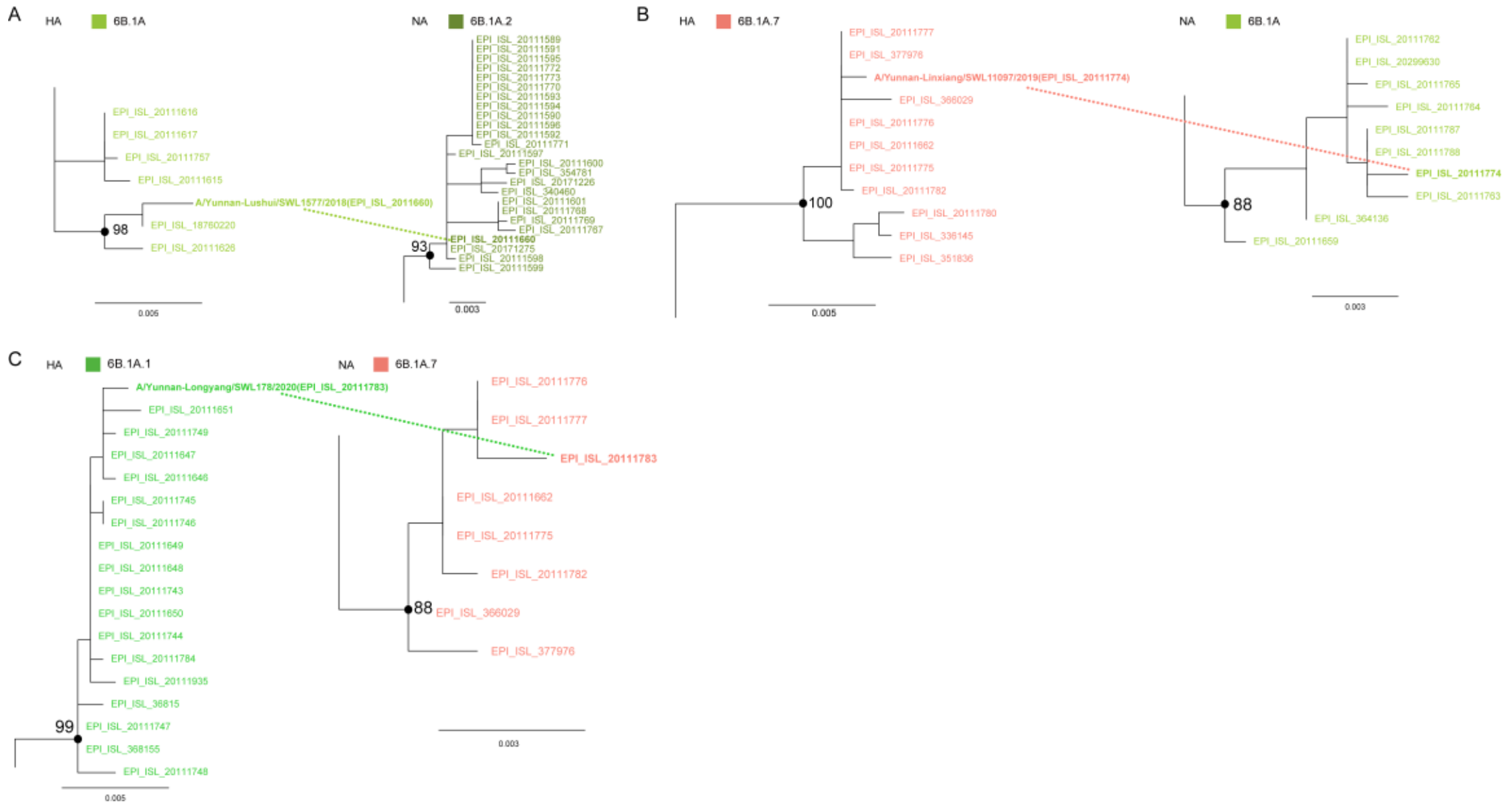

Figure S3. Three reassortment events supported by parts of HA and NA phylogenies with highly reliable bootstrap values. (A) The reassortment event occurred for the strain─A/Yunnan-Lushui/SWL1577/2018. (B) The reassortment event occurred for the strain─A/Yunnan-Linxiang/SWL11097/2019. (C) The reassortment event occurred for the strain─A/Yunnan-Longyang/SWL178/2020.

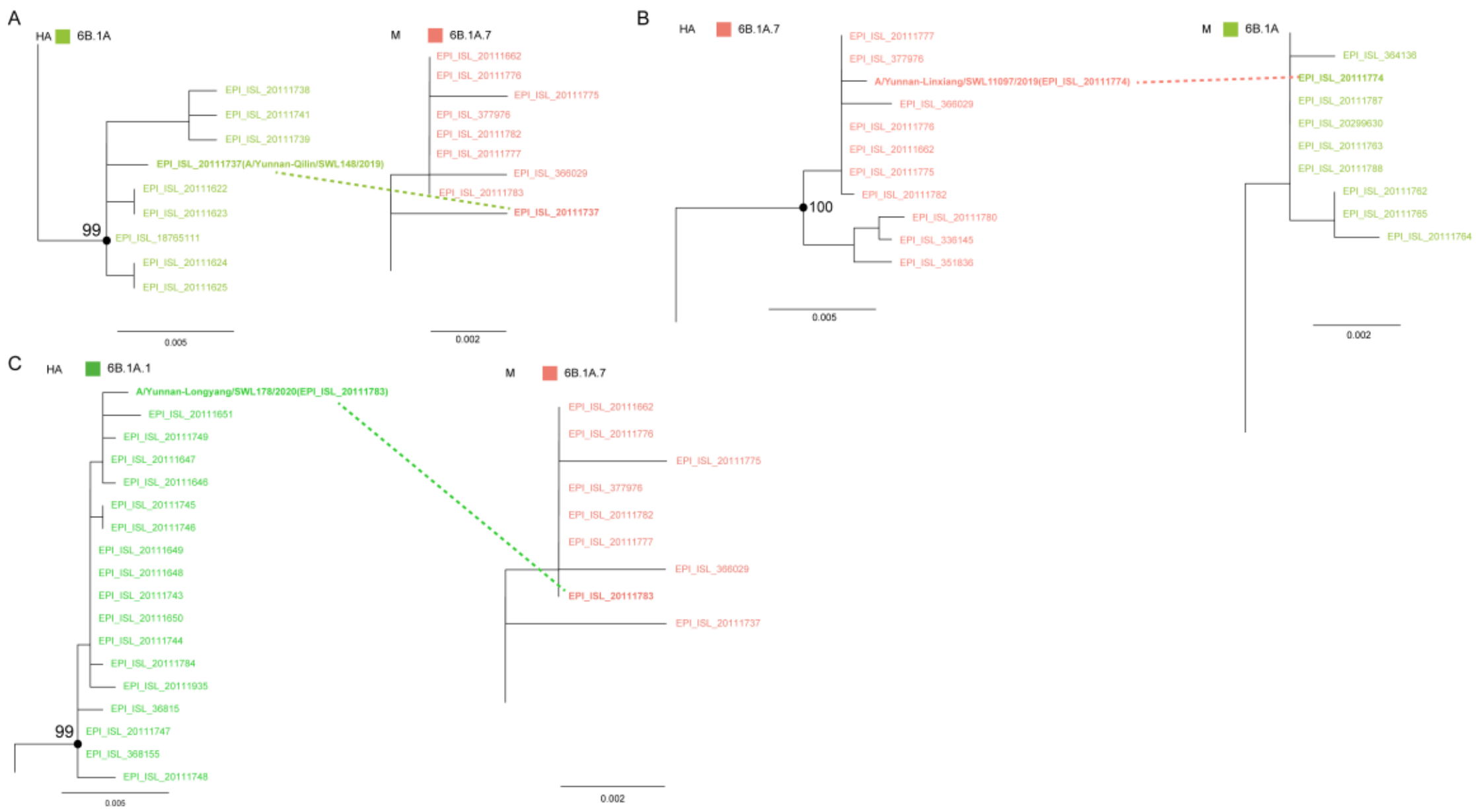

Figure S4. Three reassortment events supported by parts of HA and M phylogeny topologies. (A) The reassortment event occurred for the strain—A/Yunnan-Qilin/SWL148/2019. (B) The reassortment event occurred for the strain—A/Yunnan-Linxiang/SWL11097/2019. (C) The reassortment event occurred for the strain—A/Yunnan-Longyang/SWL178/2020.

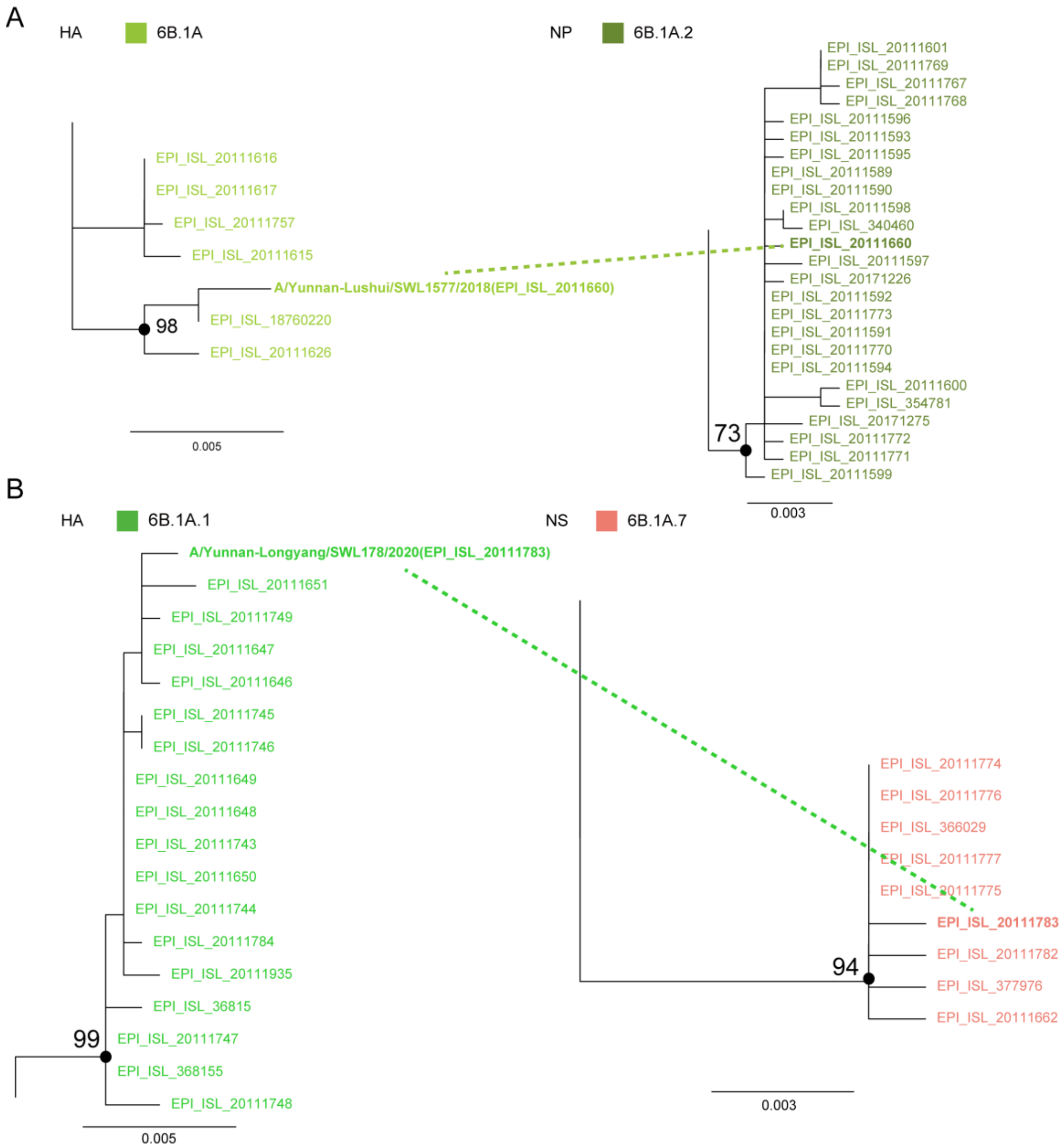

Figure S5. Reassortment events supported by parts of HA, NP, and NS phylogenies with reliable bootstrap values. (A) The reassortment event occurred for the strain—A/Yunnan-Lushui/SWL1577/2018. (B) The reassortment event occurred for the strain—A/Yunnan-Longyang/SWL178/2020.

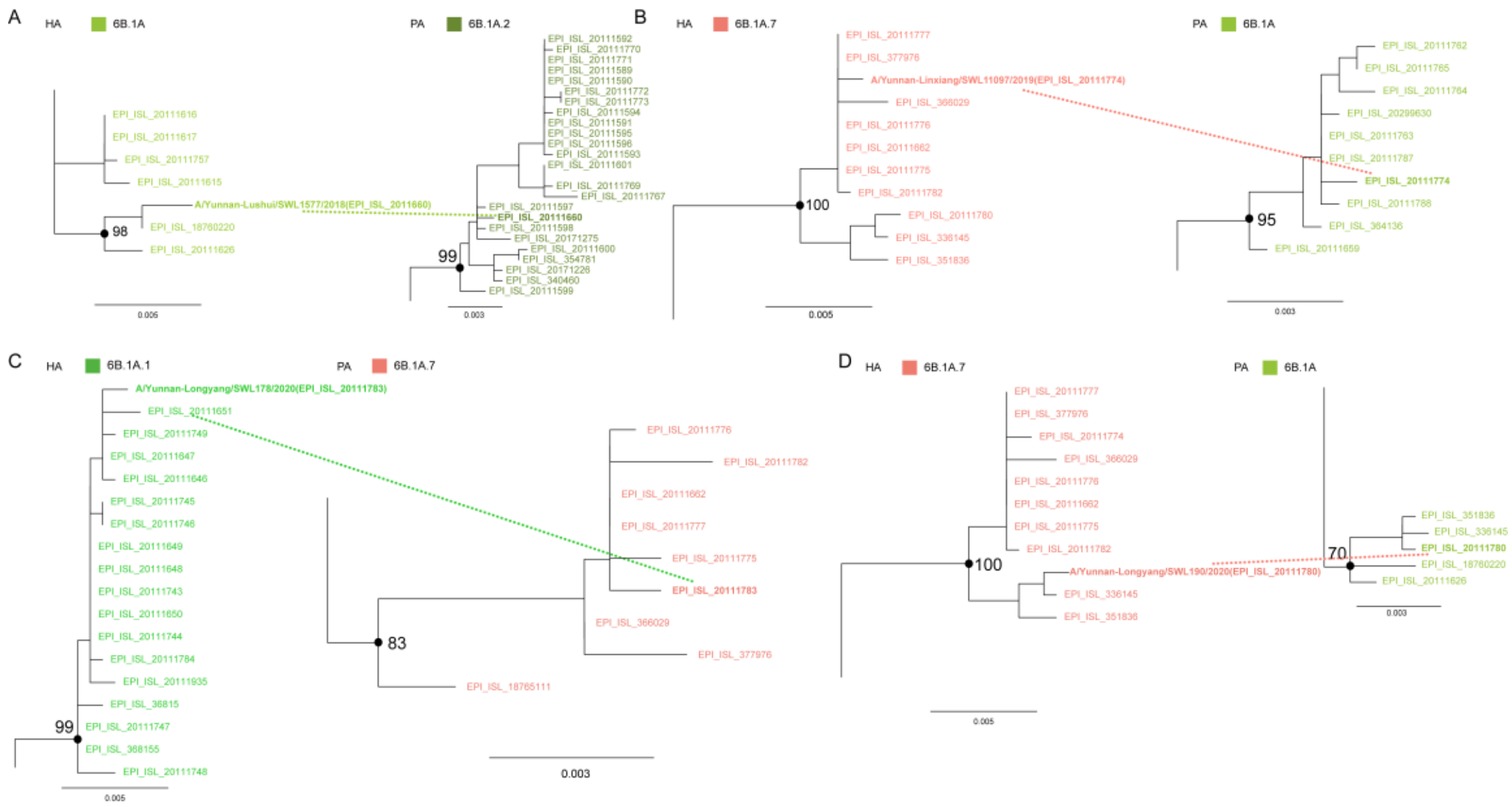

Figure S6. Three reassortment events supported by parts of *HA* and *PA* phylogenies. (A) The reassortment event occurred for the strain—A/Yunnan-Lushui/SWL1577/2018. (B) The reassortment event occurred for the strain—A/Yunnan-Linxiang/SWL11097/2019. (C) The reassortment event occurred for the strain—A/Yunnan-Longyang/SWL178/2020.(D) The reassortment event occurred for the strain—A/Yunnan-Longyang/SWL190/2020.

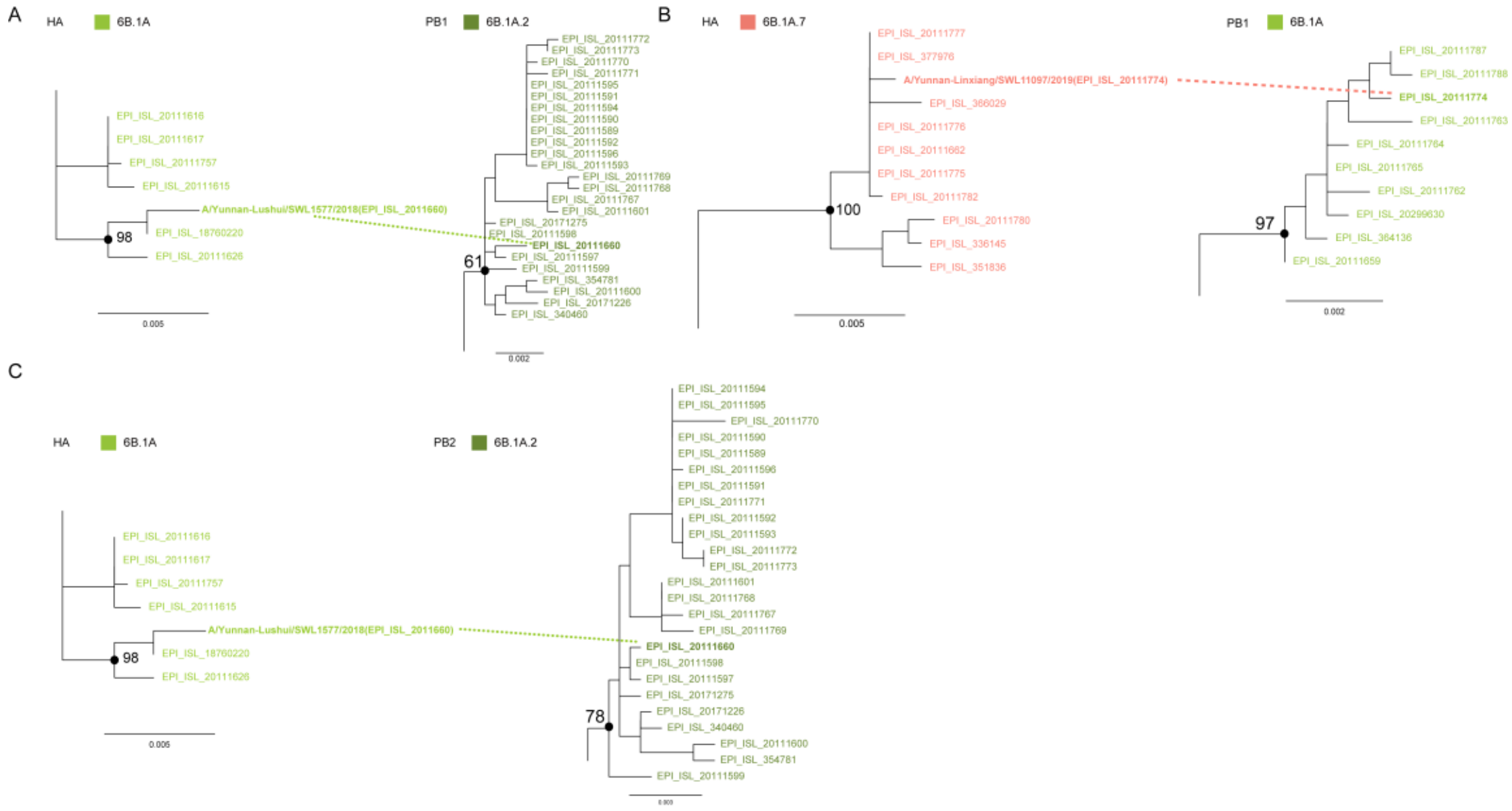

Figure S7. Reassortment events supported by parts of HA, PB1, and PB2 phylogenies with reliable bootstrap values. (A) The reassortment event occurred for the strain—A/Yunnan-Lushui/SWL1577/2018 when comparing HA and PB1 phylogenies. (B) The reassortment event occurred for the strain—A/Yunnan-Linxiang/SWL11097/2019 when comparing HA and PB1 phylogenies. (C) The reassortment event occurred for the strain—A/Yunnan-Lushui/SWL1577/2018 when comparing HA and PB2 phylogenies.

Table S1. The number (%) of influenza strains isolated from samples of influenza-like cases in Yunnan, China, from seven influenza seasons. These influenza seasons covered the samples from 1 January 2018 to 31 December 2023.

Influenza seasons	Number of samples tested	Number (%) of isolated strains				
		Type A			Type B	Total (each influenza season)
		Type A total	A(H3N2)	A(H1N1)pdm09		
2017-2018	6495	334 (5.14)	35 (10.48)	299 (89.52)	180 (2.77)	514 (7.91)
2018-2019	22588	1122 (4.97)	202 (18.00)	920 (82.00)	151 (0.67)	1273 (5.64)
2019-2020	20840	573 (2.75)	395 (68.94)	178 (31.06)	328 (1.57)	901 (4.32)
2020-2021	20099	40 (0.20)	29 (72.50)	11 (27.50)	14 (0.07)	54 (0.27)
2021-2022	27087	5 (0.02)	5 (100.00)	0 (0.00)	615 (2.27)	620 (2.29)
2022-2023	26941	963 (3.57)	620 (64.38)	343 (35.62)	49 (0.18)	1012 (3.76)
2023-2024	21266	869 (4.09)	707 (80.62)	162 (18.47)	246 (1.16)	1115 (5.24)
Total	145316	3906(2.69)	1993 (51.02)	1913 (48.98)	1583 (1.09)	5489 (3.78)

Table S2. Demographic characteristics of the cases infected with influenza viruses in Yunnan, China, from seven influenza seasons. These influenza seasons covered the samples from 1 January 2018 to 31 December 2023.

**Classification**	**Number (%) of the cases infected with influenza viruses**							
**Influenza seasons (Total cases)**	2017-2018 (514)	2018-2019 (1273)	2019-2020 (901)	2020-2021 (54)	2021-2022 (620)	2022-2023 (1012)	2023-2024 (1115)	Total (5489)
**Sex**								
female	238(46.30)	562(44.15)	399(44.28)	22(40.74)	241(38.87)	451(44.57)	548(49.15)	2461 (44.84)
male	276(53.70)	711(55.85)	502(55.72)	32(59.26)	379(61.13)	561(55.43)	567(50.85)	3028 (55.16)
**Age group (years)**								
0～	143(27.82)	358(28.12)	249(27.64)	26 (48.15)	83 (13.39)	144(14.23)	144(12.91)	1147(20.90)
5～	159(30.93)	435(34.17)	430(47.72)	12 (22.22)	235(37.90)	428(42.29)	439(39.37)	2138 (38.95)
15～	65(12.65)	174(13.67)	124(13.76)	4 (7.41)	158(25.48)	246(24.31)	241(21.61)	1012(18.44)
25～	125(24.32)	267(20.97)	86(9.54)	7(12.96)	138(22.26)	181(17.89)	266(23.86)	1070(19.49)
60～	22 (4.28)	39 (3.06)	12 (1.33)	5 (9.26)	6 (0.97)	1 3(1.28)	25 (2.24)	122 (2.22)
**Occupation**								
Nursery children	163(31.71)	323(25.37)	239(26.53)	27(50.00)	77(12.42)	171(16.90)	198(17.76)	1198(21.83)
Scattered children	58(11.28)	168(13.20)	95(10.54)	6(11.11)	36(5.81)	40(3.95)	47(4.22)	450(8.20)
Students	118(22.96)	395(31.03)	401(44.51)	4(7.41)	322(51.94)	536(52.96)	503(45.11)	2279(41.52)
Cadre and employees	22(4.28)	66(5.18)	33(3.66)	0(0.00)	22(3.55)	68(6.72)	55(4.93)	266(4.85)
Workers	13(2.53)	24(1.89)	8(0.89)	0(0.00)	21(3.39)	14(1.38)	8(0.72)	88(1.60)
Farmers	24(4.67)	83(6.52)	21(2.33)	1(1.85)	19(3.06)	28(2.77)	16(1.43)	192(3.50)
Others	116(22.57)	214(16.81)	104(11.54)	16(29.63)	123(19.84)	155(15.32)	288(25.83)	1016(18.51)

Table S3. Potential reassortment events between HA and other segments of influenza A(H1N1)pdm09 viruses circulating in Yunnan Province, China, from diverse influenza seasons. Here, the reference strains and vaccine strains in phylogenies also were included in our analyses. The reference strains with potential segment reassortment events were colored by green. The vaccine strain with potential segment reassortment event was colored by blue. The reassortment events supported by two methods including phylogeny and giraf were colored by light blue shading. And the reassortment events supported by phylogeny were colored by light pink shading.

Gisaid ID	Strain Name	HA	NA	M	NP	NS	PA	PB1	PB2
EPI_ISL_20111600	A/Yunnan-Lushui/SWL1871/2018	6B.1A.2	6B.1A.2	6B.1A.1 (tree)	6B.1A.2	6B.1A.2	6B.1A.2	6B.1A.2	6B.1A.2
EPI_ISL_20111783	A/Yunnan-Longyang/SWL178/2020	6B.1A.1	6B.1A.7 (tree+giraf)	6B.1A.7 (tree+giraf)	6B.1A.1	6B.1A.7 (tree+giraf)	6B.1A.7 (tree+giraf)	6B.1A.1	6B.1A.1
EPI_ISL_20111737	A/Yunnan-Qilin/SWL148/2019	6B.1A	6B.1A	6B.1A.7 (tree+giraf)	6B.1A	6B.1A	6B.1A	6B.1A	6B.1A
EPI_ISL_20111793	A/Yunnan-Wenshan/SWL148/2020	6B.1A.1	6B.1A.1	6B.1A (tree)	6B.1A.1	6B.1A.1	6B.1A.1	6B.1A.1	6B.1A (tree)
EPI_ISL_20111774	A/Yunnan-Linxiang/SWL11097/2019	6B.1A.7	6B.1A (tree+giraf)	6B.1A (tree+giraf)	6B.1A.7	6B.1A.7	6B.1A (tree+giraf)	6B.1A (tree+giraf)	6B.1A.7
EPI_ISL_20111660	A/Yunnan-Lushui/SWL1577/2018	6B.1A	6B.1A.2 (tree+giraf)	6B.1A	6B.1A.2 (tree+giraf)	6B.1A.2 (tree)	6B.1A.2 (tree+giraf)	6B.1A.2 (tree+giraf)	6B.1A.2 (tree+giraf)
EPI_ISL_20111780	A/Yunnan-Longyang/SWL190/2020	6B.1A.7	6B.1A.7	6B.1A.7	6B.1A.7	6B.1A.7	6B.1A (tree+giraf)	6B.1A.7	6B.1A.7
EPI_ISL_20111645	A/Yunnan-Dali/SWL1142/2018	6B.1A.1	6B.1A.1	6B.1A.1	6B.1A.1	6B.1A.1	6B.1A.1	6B.1A (tree)	6B.1A.1
EPI_ISL_2588613	A/Pennsylvania/159/2020	6B.1A.5a.1	6B.1A.5a.1	6B.1A.5a.2 (tree+giraf)	6B.1A.5a.1	6B.1A.5a.1	6B.1A.5a.2 (tree+giraf)	6B.1A.5a.1	6B.1A.5a.2 (tree+giraf)
EPI_ISL_354781	A/KANAGAWA/IC1832/2019	6B.1A.2	6B.1A.2	6B.1A.1 (tree)	6B.1A.2	6B.1A.2	6B.1A.2	6B.1A.2	6B.1A.2
EPI_ISL_20171226	A/New_Zealand/18CF0739/2018	6B.1A.2	6B.1A.2	6B.1A.2	6B.1A.2	6B.1A (tree)	6B.1A.2	6B.1A.2	6B.1A.2
EPI_ISL_353346	A/Nigeria/3626/2018	6B.1A.5b	6B.1A.5b	6B.1A.5b	6B.1A.5b	6B.1A.5b	6B.1A (tree+giraf)	6B.1A.5b	6B.1A.5b
EPI_ISL_18765111	A/South_Africa/R08500/2018	6B.1A	6B.1A	6B.1A	6B.1A	6B.1A	6B.1A.7 (tree+giraf)	6B.1A.7 (tree)	6B.1A.2 (tree）
EPI_ISL_351836	A/Pakistan/122/2019	6B.1A.7	6B.1A.7	6B.1A.7	6B.1A.7	6B.1A.7	6B.1A (tree+giraf)	6B.1A.7	6B.1A.7
EPI_ISL_330190	A/Brisbane/02/2018	6B.1A.1	6B.1A.1	6B.1A.1	6B.1A.1	6B.1A.1	6B.1A.1	6B.1A (tree）	6B.1A.1
EPI_ISL_336145	A/Eskilstuna/6/2018	6B.1A.7	6B.1A.7	6B.1A.7	6B.1A.7	6B.1A.7	6B.1A (tree+giraf)	6B.1A.7	6B.1A.7

Table S4. Selection pressures and positively selected sites of coding sequences of influenza A(H1N1)pdm09 viruses identified in Yunnan from different influenza seasons. Note: d_N/d_S, non-synonymous/synonymous substitution ratio; *p*-Value of the SLAC, MEME, and FEL results exhibiting positive selection level; N/D, not detected.

Coding region	SLAC (Single-Likelihood Ancestor Counting)			MEME (Mixed Effects Model of Evolution)			FEL (Fixed Effects Likelihood)		
	d_N/d_S	Position	*p*-Value	d_N/d_S	Position	*p*-Value	d_N/d_S	Position	*p*-Value
PB2	0.088	N/D	N/D	0.084	295	0.056	0.084	N/D	N/D
PB1	0.103	N/D	N/D	0.097	N/D	N/D	0.097	N/D	N/D
PA	0.135	N/D	N/D	0.125	N/D	N/D	0.125	N/D	N/D
HA	0.251	N/D	N/D	0.232	435	0.096	0.232	435	0.074
NP	0.104	N/D	N/D	0.095	N/D	N/D	0.095	28<br>223	0.077<br>0.094
NA	0.250	N/D	N/D	0.211	41<br>188	0.053<br>0.081	0.211	83<br>188<br>454	0.097<br>0.061<br>0.095
M1	0.072	N/D	N/D	0.068	N/D	N/D	0.068	N/D	N/D
M2	0.516	N/D	N/D	0.469	N/D	N/D	0.469	21	0.082
NS1	0.378	36	0.08	0.365	36<br>123	0.033<br>0.033	0.365	36	0.0217
NS2	0.188	N/D	N/D	0.172	N/D	N/D	0.172	N/D	N/D

Table S8. Strain names and accession numbers of eight segments of influenza A(H1N1)pdm09 viruses isolated in Yunnan Province, China, from influenza seasons. The accession IDs of 40 global reference strains, downloading from GISAID (https://gisaid.org/), also were provided in this table.

Strain name	ID	PB2	PB1	PA	HA	NP	NA	M	NS
A/Yunnan-Dali/SWL1612/2019	EPI_ISL_20111935	EPI4606243	EPI4606244	EPI4606242	EPI4606246	EPI4606239	EPI4606245	EPI4606241	EPI4606240
A/Yunnan-Anning/SWL1128/2023	EPI_ISL_20111933	EPI4606235	EPI4606236	EPI4606234	EPI4606238	EPI4606231	EPI4606237	EPI4606233	EPI4606232
A/Yunnan-Xishan/SWL1410/2023	EPI_ISL_20111932	EPI4606227	EPI4606228	EPI4606226	EPI4606230	EPI4606223	EPI4606229	EPI4606225	EPI4606224
A/Yunnan-Mangshi/SWL1565/2023	EPI_ISL_20111931	EPI4606219	EPI4606220	EPI4606218	EPI4606222	EPI4606215	EPI4606221	EPI4606217	EPI4606216
A/Yunnan-Zhaoyang/SWL1572/2023	EPI_ISL_20111930	EPI4606211	EPI4606212	EPI4606210	EPI4606214	EPI4606207	EPI4606213	EPI4606209	EPI4606208
A/Yunnan-Wuhua/SWL1397/2023	EPI_ISL_20111929	EPI4606203	EPI4606204	EPI4606202	EPI4606206	EPI4606199	EPI4606205	EPI4606201	EPI4606200
A/Yunnan-Xianggelila/SWL1986/2023	EPI_ISL_20111928	EPI4606195	EPI4606196	EPI4606194	EPI4606198	EPI4606191	EPI4606197	EPI4606193	EPI4606192
A/Yunnan-Mangshi/SWL1561/2023	EPI_ISL_20111927	EPI4606187	EPI4606188	EPI4606186	EPI4606190	EPI4606183	EPI4606189	EPI4606185	EPI4606184
A/Yunnan-Mangshi/SWL1449/2023	EPI_ISL_20111926	EPI4606179	EPI4606180	EPI4606178	EPI4606182	EPI4606175	EPI4606181	EPI4606177	EPI4606176
A/Yunnan-Mangshi/SWL1430/2023	EPI_ISL_20111925	EPI4606171	EPI4606172	EPI4606170	EPI4606174	EPI4606167	EPI4606173	EPI4606169	EPI4606168
A/Yunnan-Anning/SWL1145/2023	EPI_ISL_20111924	EPI4606163	EPI4606164	EPI4606162	EPI4606166	EPI4606159	EPI4606165	EPI4606161	EPI4606160
A/Yunnan-Linxiang/SWL1424/2023	EPI_ISL_20111923	EPI4606155	EPI4606156	EPI4606154	EPI4606158	EPI4606151	EPI4606157	EPI4606153	EPI4606152
A/Yunnan-Guandu/SWL140/2023	EPI_ISL_20111922	EPI4606147	EPI4606148	EPI4606146	EPI4606150	EPI4606143	EPI4606149	EPI4606145	EPI4606144
A/Yunnan-Wenshan/SWL1328/2023	EPI_ISL_20111921	EPI4606139	EPI4606140	EPI4606138	EPI4606142	EPI4606135	EPI4606141	EPI4606137	EPI4606136
A/Yunnan-Mangshi/SWL1579/2023	EPI_ISL_20111920	EPI4606131	EPI4606132	EPI4606130	EPI4606134	EPI4606127	EPI4606133	EPI4606129	EPI4606128
A/Yunnan-Zhaoyang/SWL1366/2023	EPI_ISL_20111919	EPI4606123	EPI4606124	EPI4606122	EPI4606126	EPI4606119	EPI4606125	EPI4606121	EPI4606120
A/Yunnan-Mangshi/SWL1589/2023	EPI_ISL_20111918	EPI4606115	EPI4606116	EPI4606114	EPI4606118	EPI4606111	EPI4606117	EPI4606113	EPI4606112
A/Yunnan-Jinghong/SWL1522/2023	EPI_ISL_20111917	EPI4606107	EPI4606108	EPI4606106	EPI4606110	EPI4606103	EPI4606109	EPI4606105	EPI4606104
A/Yunnan-Dali/SWL1333/2023	EPI_ISL_20111916	EPI4606099	EPI4606100	EPI4606098	EPI4606102	EPI4606095	EPI4606101	EPI4606097	EPI4606096
A/Yunnan-Linxiang/SWL1590/2023	EPI_ISL_20111915	EPI4606091	EPI4606092	EPI4606090	EPI4606094	EPI4606087	EPI4606093	EPI4606089	EPI4606088
A/Yunnan-Wenshan/SWL1313/2023	EPI_ISL_20111914	EPI4606083	EPI4606084	EPI4606082	EPI4606086	EPI4606079	EPI4606085	EPI4606081	EPI4606080
A/Yunnan-Zhaoyang/SWL1567/2023	EPI_ISL_20111913	EPI4606075	EPI4606076	EPI4606074	EPI4606078	EPI4606071	EPI4606077	EPI4606073	EPI4606072
A/Yunnan-Zhaoyang/SWL1545/2023	EPI_ISL_20111912	EPI4606067	EPI4606068	EPI4606066	EPI4606070	EPI4606063	EPI4606069	EPI4606065	EPI4606064
A/Yunnan-Zhaoyang/SWL1253/2023	EPI_ISL_20111911	EPI4606059	EPI4606060	EPI4606058	EPI4606062	EPI4606055	EPI4606061	EPI4606057	EPI4606056
A/Yunnan-Mangshi/SWL1513/2023	EPI_ISL_20111910	EPI4606051	EPI4606052	EPI4606050	EPI4606054	EPI4606047	EPI4606053	EPI4606049	EPI4606048
A/Yunnan-Mangshi/SWL1401/2023	EPI_ISL_20111909	EPI4606043	EPI4606044	EPI4606042	EPI4606046	EPI4606039	EPI4606045	EPI4606041	EPI4606040
A/Yunnan-Xianggelila/SWL1977/2023	EPI_ISL_20111908	EPI4606035	EPI4606036	EPI4606034	EPI4606038	EPI4606031	EPI4606037	EPI4606033	EPI4606032
A/Yunnan-Lushui/SWL1200/2023	EPI_ISL_20111907	EPI4606027	EPI4606028	EPI4606026	EPI4606030	EPI4606023	EPI4606029	EPI4606025	EPI4606024
A/Yunnan-Lushui/SWL1537/2023	EPI_ISL_20111906	EPI4606019	EPI4606020	EPI4606018	EPI4606022	EPI4606015	EPI4606021	EPI4606017	EPI4606016
A/Yunnan-Guandu/SWL350/2023	EPI_ISL_20111905	EPI4606011	EPI4606012	EPI4606010	EPI4606014	EPI4606007	EPI4606013	EPI4606009	EPI4606008
A/Yunnan-Dali/SWL1299/2023	EPI_ISL_20111904	EPI4606003	EPI4606004	EPI4606002	EPI4606006	EPI4605999	EPI4606005	EPI4606001	EPI4606000
A/Yunnan-Dali/SWL1325/2023	EPI_ISL_20111902	EPI4605995	EPI4605996	EPI4605994	EPI4605998	EPI4605991	EPI4605997	EPI4605993	EPI4605992
A/Yunnan-Mangshi/SWL1443/2023	EPI_ISL_20111901	EPI4605987	EPI4605988	EPI4605986	EPI4605990	EPI4605983	EPI4605989	EPI4605985	EPI4605984
A/Yunnan-Chuxiong/SWL313/2023	EPI_ISL_20111900	EPI4605979	EPI4605980	EPI4605978	EPI4605982	EPI4605975	EPI4605981	EPI4605977	EPI4605976
A/Yunnan-Chuxiong/SWL1159/2023	EPI_ISL_20111899	EPI4605971	EPI4605972	EPI4605970	EPI4605974	EPI4605967	EPI4605973	EPI4605969	EPI4605968
A/Yunnan-Chuxiong/SWL1150/2023	EPI_ISL_20111898	EPI4605963	EPI4605964	EPI4605962	EPI4605966	EPI4605959	EPI4605965	EPI4605961	EPI4605960
A/Yunnan-Jinghong/SWL1525/2023	EPI_ISL_20111897	EPI4605955	EPI4605956	EPI4605954	EPI4605958	EPI4605951	EPI4605957	EPI4605953	EPI4605952
A/Yunnan-Xianggelila/SWL331/2023	EPI_ISL_20111896	EPI4605947	EPI4605948	EPI4605946	EPI4605950	EPI4605943	EPI4605949	EPI4605945	EPI4605944
A/Yunnan-Mangshi/SWL1478/2023	EPI_ISL_20111895	EPI4605939	EPI4605940	EPI4605938	EPI4605942	EPI4605935	EPI4605941	EPI4605937	EPI4605936

A/Yunnan-Longyang/SWL1790/2023	EPI_ISL_20111894	EPI4605931	EPI4605932	EPI4605930	EPI4605934	EPI4605927	EPI4605933	EPI4605929	EPI4605928
A/Yunnan-Linxiang/SWL1502/2023	EPI_ISL_20111893	EPI4605923	EPI4605924	EPI4605922	EPI4605926	EPI4605919	EPI4605925	EPI4605921	EPI4605920
A/Yunnan-Dali/SWL1312/2023	EPI_ISL_20111892	EPI4605915	EPI4605916	EPI4605914	EPI4605918	EPI4605911	EPI4605917	EPI4605913	EPI4605912
A/Yunnan-Huize/SWL32/2023	EPI_ISL_20111891	EPI4605907	EPI4605908	EPI4605906	EPI4605910	EPI4605903	EPI4605909	EPI4605905	EPI4605904
A/Yunnan-Qilin/SWL1292/2023	EPI_ISL_20111890	EPI4605899	EPI4605900	EPI4605898	EPI4605902	EPI4605895	EPI4605901	EPI4605897	EPI4605896
A/Yunnan-Mangshi/SWL1596/2023	EPI_ISL_20111876	EPI4605891	EPI4605892	EPI4605890	EPI4605894	EPI4605887	EPI4605893	EPI4605889	EPI4605888
A/Yunnan-Mangshi/SWL1547/2023	EPI_ISL_20111875	EPI4605883	EPI4605884	EPI4605882	EPI4605886	EPI4605879	EPI4605885	EPI4605881	EPI4605880
A/Yunnan-Linxiang/SWL1798/2023	EPI_ISL_20111874	EPI4605875	EPI4605876	EPI4605874	EPI4605878	EPI4605871	EPI4605877	EPI4605873	EPI4605872
A/Yunnan-Gucheng/SWL1230/2023	EPI_ISL_20111872	EPI4605867	EPI4605868	EPI4605866	EPI4605870	EPI4605863	EPI4605869	EPI4605865	EPI4605864
A/Yunnan-Lushui/SWL1588/2023	EPI_ISL_20111871	EPI4605859	EPI4605860	EPI4605858	EPI4605862	EPI4605855	EPI4605861	EPI4605857	EPI4605856
A/Yunnan-Lushui/SWL1659/2023	EPI_ISL_20111870	EPI4605851	EPI4605852	EPI4605850	EPI4605854	EPI4605847	EPI4605853	EPI4605849	EPI4605848
A/Yunnan-Lushui/SWL1579/2023	EPI_ISL_20111869	EPI4605843	EPI4605844	EPI4605842	EPI4605846	EPI4605839	EPI4605845	EPI4605841	EPI4605840
A/Yunnan-Lushui/SWL1338/2023	EPI_ISL_20111868	EPI4605835	EPI4605836	EPI4605834	EPI4605838	EPI4605831	EPI4605837	EPI4605833	EPI4605832
A/Yunnan-Lushui/SWL1711/2023	EPI_ISL_20111867	EPI4605827	EPI4605828	EPI4605826	EPI4605830	EPI4605823	EPI4605829	EPI4605825	EPI4605824
A/Yunnan-Lushui/SWL1318/2023	EPI_ISL_20111866	EPI4605819	EPI4605820	EPI4605818	EPI4605822	EPI4605815	EPI4605821	EPI4605817	EPI4605816
A/Yunnan-Mengzi/SWL199/2023	EPI_ISL_20111865	EPI4605811	EPI4605812	EPI4605810	EPI4605814	EPI4605807	EPI4605813	EPI4605809	EPI4605808
A/Yunnan-Xianggelila/SWL1282/2023	EPI_ISL_20111864	EPI4605803	EPI4605804	EPI4605802	EPI4605806	EPI4605799	EPI4605805	EPI4605801	EPI4605800
A/Yunnan-Xishan/SWL1391/2023	EPI_ISL_20111863	EPI4605795	EPI4605796	EPI4605794	EPI4605798	EPI4605791	EPI4605797	EPI4605793	EPI4605792
A/Yunnan-Xishan/SWL1344/2023	EPI_ISL_20111862	EPI4605787	EPI4605788	EPI4605786	EPI4605790	EPI4605783	EPI4605789	EPI4605785	EPI4605784
A/Yunnan-Hongta/SWL11986/2023	EPI_ISL_20111861	EPI4605779	EPI4605780	EPI4605778	EPI4605782	EPI4605775	EPI4605781	EPI4605777	EPI4605776
A/Yunnan-Xishan/SWL1321/2023	EPI_ISL_20111860	EPI4605771	EPI4605772	EPI4605770	EPI4605774	EPI4605767	EPI4605773	EPI4605769	EPI4605768
A/Yunnan-Wuhua/SWL1177/2023	EPI_ISL_20111858	EPI4605763	EPI4605764	EPI4605762	EPI4605766	EPI4605759	EPI4605765	EPI4605761	EPI4605760
A/Yunnan-Linxiang/SWL1581/2023	EPI_ISL_20111857	EPI4605755	EPI4605756	EPI4605754	EPI4605758	EPI4605751	EPI4605757	EPI4605753	EPI4605752
A/Yunnan-Wuhua/SWL1136/2023	EPI_ISL_20111856	EPI4605747	EPI4605748	EPI4605746	EPI4605750	EPI4605743	EPI4605749	EPI4605745	EPI4605744
A/Yunnan-Anning/SWL1133/2023	EPI_ISL_20111836	EPI4605739	EPI4605740	EPI4605738	EPI4605742	EPI4605735	EPI4605741	EPI4605737	EPI4605736
A/Yunnan-Jinghong/SWL1515/2023	EPI_ISL_20111835	EPI4605731	EPI4605732	EPI4605730	EPI4605734	EPI4605727	EPI4605733	EPI4605729	EPI4605728
A/Yunnan-Linxiang/SWL1235/2023	EPI_ISL_20111834	EPI4605723	EPI4605724	EPI4605722	EPI4605726	EPI4605719	EPI4605725	EPI4605721	EPI4605720
A/Yunnan-Linxiang/SWL1255/2023	EPI_ISL_20111833	EPI4605715	EPI4605716	EPI4605714	EPI4605718	EPI4605711	EPI4605717	EPI4605713	EPI4605712
A/Yunnan-Guandu/SWL322/2023	EPI_ISL_20111832	EPI4605707	EPI4605708	EPI4605706	EPI4605710	EPI4605703	EPI4605709	EPI4605705	EPI4605704
A/Yunnan-Guandu/SWL339/2023	EPI_ISL_20111831	EPI4605699	EPI4605700	EPI4605698	EPI4605702	EPI4605695	EPI4605701	EPI4605697	EPI4605696
A/Yunnan-Dali/SWL1293/2023	EPI_ISL_20111830	EPI4605691	EPI4605692	EPI4605690	EPI4605694	EPI4605687	EPI4605693	EPI4605689	EPI4605688
A/Yunnan-Zhaoyang/SWL1560/2023	EPI_ISL_20111829	EPI4605683	EPI4605684	EPI4605682	EPI4605686	EPI4605679	EPI4605685	EPI4605681	EPI4605680
A/Yunnan-Zhaoyang/SWL1373/2023	EPI_ISL_20111828	EPI4605675	EPI4605676	EPI4605674	EPI4605678	EPI4605671	EPI4605677	EPI4605673	EPI4605672
A/Yunnan-Linxiang/SWL1800/2023	EPI_ISL_20111827	EPI4605667	EPI4605668	EPI4605666	EPI4605670	EPI4605663	EPI4605669	EPI4605665	EPI4605664
A/Yunnan-Linxiang/SWL1585/2023	EPI_ISL_20111826	EPI4605659	EPI4605660	EPI4605658	EPI4605662	EPI4605655	EPI4605661	EPI4605657	EPI4605656
A/Yunnan-Linxiang/SWL1511/2023	EPI_ISL_20111825	EPI4605651	EPI4605652	EPI4605650	EPI4605654	EPI4605647	EPI4605653	EPI4605649	EPI4605648
A/Yunnan-Zhaoyang/SWL1160/2023	EPI_ISL_20111824	EPI4605643	EPI4605644	EPI4605642	EPI4605646	EPI4605639	EPI4605645	EPI4605641	EPI4605640
A/Yunnan-Anning/SWL1161/2023	EPI_ISL_20111823	EPI4605635	EPI4605636	EPI4605634	EPI4605638	EPI4605631	EPI4605637	EPI4605633	EPI4605632
A/Yunnan-Hongta/SWL1550/2023	EPI_ISL_20111822	EPI4605627	EPI4605628	EPI4605626	EPI4605630	EPI4605623	EPI4605629	EPI4605625	EPI4605624
A/Yunnan-Hongta/SWL1552/2023	EPI_ISL_20111821	EPI4605619	EPI4605620	EPI4605618	EPI4605622	EPI4605615	EPI4605621	EPI4605617	EPI4605616
A/Yunnan-Linxiang/SWL1427/2023	EPI_ISL_20111820	EPI4605611	EPI4605612	EPI4605610	EPI4605614	EPI4605607	EPI4605613	EPI4605609	EPI4605608
A/Yunnan-Xianggelila/SWL1232/2023	EPI_ISL_20111819	EPI4605603	EPI4605604	EPI4605602	EPI4605606	EPI4605599	EPI4605605	EPI4605601	EPI4605600
A/Yunnan-Lufeng/SWL310/2023	EPI_ISL_20111818	EPI4605595	EPI4605596	EPI4605594	EPI4605598	EPI4605591	EPI4605597	EPI4605593	EPI4605592
A/Yunnan-Xianggelila/SWL1980/2023	EPI_ISL_20111817	EPI4605587	EPI4605588	EPI4605586	EPI4605590	EPI4605583	EPI4605589	EPI4605585	EPI4605584

A/Yunnan-Xianggelila/SWL1295/2023	EPI_ISL_20111816	EPI4605579	EPI4605580	EPI4605578	EPI4605582	EPI4605575	EPI4605581	EPI4605577	EPI4605576
A/Yunnan-Longyang/SWL1631/2023	EPI_ISL_20111815	EPI4605571	EPI4605572	EPI4605570	EPI4605574	EPI4605567	EPI4605573	EPI4605569	EPI4605568
A/Yunnan-Linxiang/SWL131/2021	EPI_ISL_20111814	EPI4605563	EPI4605564	EPI4605562	EPI4605566	EPI4605559	EPI4605565	EPI4605561	EPI4605560
A/Yunnan-Wuhua/SWL191/2020	EPI_ISL_20111813	EPI4605555	EPI4605556	EPI4605554	EPI4605558	EPI4605551	EPI4605557	EPI4605553	EPI4605552
A/Yunnan-Mangshi/SWL164/2020	EPI_ISL_20111812	EPI4605547	EPI4605548	EPI4605546	EPI4605550	EPI4605543	EPI4605549	EPI4605545	EPI4605544
A/Yunnan-Wuhua/SWL175/2020	EPI_ISL_20111811	EPI4605539	EPI4605540	EPI4605538	EPI4605542	EPI4605535	EPI4605541	EPI4605537	EPI4605536
A/Yunnan-Xianggelila/SWL1248/2020	EPI_ISL_20111810	EPI4605531	EPI4605532	EPI4605530	EPI4605534	EPI4605527	EPI4605533	EPI4605529	EPI4605528
A/Yunnan-Gucheng/SWL1276/2020	EPI_ISL_20111809	EPI4605523	EPI4605524	EPI4605522	EPI4605526	EPI4605519	EPI4605525	EPI4605521	EPI4605520
A/Yunnan-Xianggelila/SWL1263/2020	EPI_ISL_20111808	EPI4605515	EPI4605516	EPI4605514	EPI4605518	EPI4605511	EPI4605517	EPI4605513	EPI4605512
A/Yunnan-Gucheng/SWL1286/2020	EPI_ISL_20111807	EPI4605507	EPI4605508	EPI4605506	EPI4605510	EPI4605503	EPI4605509	EPI4605505	EPI4605504
A/Yunnan-Xianggelila/SWL1219/2020	EPI_ISL_20111806	EPI4605499	EPI4605500	EPI4605498	EPI4605502	EPI4605495	EPI4605501	EPI4605497	EPI4605496
A/Yunnan-Xianggelila/SWL1268/2020	EPI_ISL_20111805	EPI4605491	EPI4605492	EPI4605490	EPI4605494	EPI4605487	EPI4605493	EPI4605489	EPI4605488
A/Yunnan-Xianggelila/SWL1277/2020	EPI_ISL_20111804	EPI4605483	EPI4605484	EPI4605482	EPI4605486	EPI4605479	EPI4605485	EPI4605481	EPI4605480
A/Yunnan-Xianggelila/SWL1284/2020	EPI_ISL_20111803	EPI4605475	EPI4605476	EPI4605474	EPI4605478	EPI4605471	EPI4605477	EPI4605473	EPI4605472
A/Yunnan-Linxiang/SWL1486/2020	EPI_ISL_20111802	EPI4605467	EPI4605468	EPI4605466	EPI4605470	EPI4605463	EPI4605469	EPI4605465	EPI4605464
A/Yunnan-Simao/SWL1113/2020	EPI_ISL_20111801	EPI4605459	EPI4605460	EPI4605458	EPI4605462	EPI4605455	EPI4605461	EPI4605457	EPI4605456
A/Yunnan-Linxiang/SWL1485/2020	EPI_ISL_20111799	EPI4605451	EPI4605452	EPI4605450	EPI4605454	EPI4605447	EPI4605453	EPI4605449	EPI4605448
A/Yunnan-Linxiang/SWL1674/2020	EPI_ISL_20111798	EPI4605443	EPI4605444	EPI4605442	EPI4605446	EPI4605439	EPI4605445	EPI4605441	EPI4605440
A/Yunnan-Linxiang/SWL1482/2020	EPI_ISL_20111797	EPI4605435	EPI4605436	EPI4605434	EPI4605438	EPI4605431	EPI4605437	EPI4605433	EPI4605432
A/Yunnan-Chuxiong/SWL1206/2020	EPI_ISL_20111796	EPI4605427	EPI4605428	EPI4605426	EPI4605430	EPI4605423	EPI4605429	EPI4605425	EPI4605424
A/Yunnan-Linxiang/SWL1661/2020	EPI_ISL_20111795	EPI4605419	EPI4605420	EPI4605418	EPI4605422	EPI4605415	EPI4605421	EPI4605417	EPI4605416
A/Yunnan-Chuxiong/SWL1207/2020	EPI_ISL_20111794	EPI4605411	EPI4605412	EPI4605410	EPI4605414	EPI4605407	EPI4605413	EPI4605409	EPI4605408
A/Yunnan-Wenshan/SWL148/2020	EPI_ISL_20111793	EPI4605403	EPI4605404	EPI4605402	EPI4605406	EPI4605399	EPI4605405	EPI4605401	EPI4605400
A/Yunnan-Simao/SWL182/2020	EPI_ISL_20111792	EPI4605395	EPI4605396	EPI4605394	EPI4605398	EPI4605391	EPI4605397	EPI4605393	EPI4605392
A/Yunnan-Linxiang/SWL1484/2020	EPI_ISL_20111791	EPI4605387	EPI4605388	EPI4605386	EPI4605390	EPI4605383	EPI4605389	EPI4605385	EPI4605384
A/Yunnan-Linxiang/SWL1483/2020	EPI_ISL_20111790	EPI4605379	EPI4605380	EPI4605378	EPI4605382	EPI4605375	EPI4605381	EPI4605377	EPI4605376
A/Yunnan-Simao/SWL1106/2020	EPI_ISL_20111789	EPI4605371	EPI4605372	EPI4605370	EPI4605374	EPI4605367	EPI4605373	EPI4605369	EPI4605368
A/Yunnan-Linxiang/SWL1664/2020	EPI_ISL_20111788	EPI4605363	EPI4605364	EPI4605362	EPI4605366	EPI4605359	EPI4605365	EPI4605361	EPI4605360
A/Yunnan-Linxiang/SWL1481/2020	EPI_ISL_20111787	EPI4605355	EPI4605356	EPI4605354	EPI4605358	EPI4605351	EPI4605357	EPI4605353	EPI4605352
A/Yunnan-Gucheng/SWL124/2020	EPI_ISL_20111786	EPI4605347	EPI4605348	EPI4605346	EPI4605350	EPI4605343	EPI4605349	EPI4605345	EPI4605344
A/Yunnan-Gucheng/SWL131/2020	EPI_ISL_20111785	EPI4605339	EPI4605340	EPI4605338	EPI4605342	EPI4605335	EPI4605341	EPI4605337	EPI4605336
A/Yunnan-Linxiang/SWL1675/2020	EPI_ISL_20111784	EPI4605331	EPI4605332	EPI4605330	EPI4605334	EPI4605327	EPI4605333	EPI4605329	EPI4605328
A/Yunnan-Longyang/SWL178/2020	EPI_ISL_20111783	EPI4605323	EPI4605324	EPI4605322	EPI4605326	EPI4605319	EPI4605325	EPI4605321	EPI4605320
A/Yunnan-Simao/SWL173/2020	EPI_ISL_20111782	EPI4605315	EPI4605316	EPI4605314	EPI4605318	EPI4605311	EPI4605317	EPI4605313	EPI4605312
A/Yunnan-Longyang/SWL190/2020	EPI_ISL_20111780	EPI4605307	EPI4605308	EPI4605306	EPI4605310	EPI4605303	EPI4605309	EPI4605305	EPI4605304
A/Yunnan-Qilin/SWL530/2019	EPI_ISL_20111779	EPI4605299	EPI4605300	EPI4605298	EPI4605302	EPI4605295	EPI4605301	EPI4605297	EPI4605296
A/Yunnan-Linxiang/SWL115/2019	EPI_ISL_20111778	EPI4605291	EPI4605292	EPI4605290	EPI4605294	EPI4605287	EPI4605293	EPI4605289	EPI4605288
A/Yunnan-Lushui/SWL119/2019	EPI_ISL_20111777	EPI4605283	EPI4605284	EPI4605282	EPI4605286	EPI4605279	EPI4605285	EPI4605281	EPI4605280
A/Yunnan-Lushui/SWL123/2019	EPI_ISL_20111776	EPI4605275	EPI4605276	EPI4605274	EPI4605278	EPI4605271	EPI4605277	EPI4605273	EPI4605272
A/Yunnan-Lushui/SWL15/2019	EPI_ISL_20111775	EPI4605267	EPI4605268	EPI4605266	EPI4605270	EPI4605263	EPI4605269	EPI4605265	EPI4605264
A/Yunnan-Linxiang/SWL11097/2019	EPI_ISL_20111774	EPI4605259	EPI4605260	EPI4605258	EPI4605262	EPI4605255	EPI4605261	EPI4605257	EPI4605256
A/Yunnan-Lushui/SWL11/2019	EPI_ISL_20111773	EPI4605251	EPI4605252	EPI4605250	EPI4605254	EPI4605247	EPI4605253	EPI4605249	EPI4605248
A/Yunnan-Lushui/SWL142/2019	EPI_ISL_20111772	EPI4605243	EPI4605244	EPI4605242	EPI4605246	EPI4605239	EPI4605245	EPI4605241	EPI4605240
A/Yunnan-Wenshan/SWL121/2019	EPI_ISL_20111771	EPI4605235	EPI4605236	EPI4605234	EPI4605238	EPI4605231	EPI4605237	EPI4605233	EPI4605232

A/Yunnan-Xiangyun/SWL35/2019	EPI_ISL_20111770	EPI4605227	EPI4605228	EPI4605226	EPI4605230	EPI4605223	EPI4605229	EPI4605225	EPI4605224
A/Yunnan-Linxiang/SWL11099/2019	EPI_ISL_20111769	EPI4605219	EPI4605220	EPI4605218	EPI4605222	EPI4605215	EPI4605221	EPI4605217	EPI4605216
A/Yunnan-Anning/SWL17/2019	EPI_ISL_20111768	EPI4605211	EPI4605212	EPI4605210	EPI4605214	EPI4605207	EPI4605213	EPI4605209	EPI4605208
A/Yunnan-Dali/SWL1613/2019	EPI_ISL_20111767	EPI4605203	EPI4605204	EPI4605202	EPI4605206	EPI4605199	EPI4605205	EPI4605201	EPI4605200
A/Yunnan-Zhaoyang/SWL123/2019	EPI_ISL_20111765	EPI4605195	EPI4605196	EPI4605194	EPI4605198	EPI4605191	EPI4605197	EPI4605193	EPI4605192
A/Yunnan-Zhaoyang/SWL111/2019	EPI_ISL_20111764	EPI4605187	EPI4605188	EPI4605186	EPI4605190	EPI4605183	EPI4605189	EPI4605185	EPI4605184
A/Yunnan-Linxiang/SWL1859/2019	EPI_ISL_20111763	EPI4605179	EPI4605180	EPI4605178	EPI4605182	EPI4605175	EPI4605181	EPI4605177	EPI4605176
A/Yunnan-Zhaoyang/SWL1182/2019	EPI_ISL_20111762	EPI4605171	EPI4605172	EPI4605170	EPI4605174	EPI4605167	EPI4605173	EPI4605169	EPI4605168
A/Yunnan-Qilin/SWL523/2019	EPI_ISL_20111761	EPI4605163	EPI4605164	EPI4605162	EPI4605166	EPI4605159	EPI4605165	EPI4605161	EPI4605160
A/Yunnan-Wenshan/SWL152/2019	EPI_ISL_20111760	EPI4605155	EPI4605156	EPI4605154	EPI4605158	EPI4605151	EPI4605157	EPI4605153	EPI4605152
A/Yunnan-Qilin/SWL57/2019	EPI_ISL_20111759	EPI4605147	EPI4605148	EPI4605146	EPI4605150	EPI4605143	EPI4605149	EPI4605145	EPI4605144
A/Yunnan-Qilin/SWL515/2019	EPI_ISL_20111758	EPI4605139	EPI4605140	EPI4605138	EPI4605142	EPI4605135	EPI4605141	EPI4605137	EPI4605136
A/Yunnan-Jinghong/SWL162/2019	EPI_ISL_20111757	EPI4605131	EPI4605132	EPI4605130	EPI4605134	EPI4605127	EPI4605133	EPI4605129	EPI4605128
A/Yunnan-Linxiang/SWL133/2019	EPI_ISL_20111756	EPI4605123	EPI4605124	EPI4605122	EPI4605126	EPI4605119	EPI4605125	EPI4605121	EPI4605120
A/Yunnan-Linxiang/SWL131/2019	EPI_ISL_20111755	EPI4605115	EPI4605116	EPI4605114	EPI4605118	EPI4605111	EPI4605117	EPI4605113	EPI4605112
A/Yunnan-Linxiang/SWL16/2019	EPI_ISL_20111754	EPI4605107	EPI4605108	EPI4605106	EPI4605110	EPI4605103	EPI4605109	EPI4605105	EPI4605104
A/Yunnan-Linxiang/SWL124/2019	EPI_ISL_20111753	EPI4605099	EPI4605100	EPI4605098	EPI4605102	EPI4605095	EPI4605101	EPI4605097	EPI4605096
A/Yunnan-Linxiang/SWL1546/2019	EPI_ISL_20111752	EPI4605091	EPI4605092	EPI4605090	EPI4605094	EPI4605087	EPI4605093	EPI4605089	EPI4605088
A/Yunnan-Linxiang/SWL1549/2019	EPI_ISL_20111750	EPI4605083	EPI4605084	EPI4605082	EPI4605086	EPI4605079	EPI4605085	EPI4605081	EPI4605080
A/Yunnan-Hongta/SWL163/2019	EPI_ISL_20111749	EPI4605075	EPI4605076	EPI4605074	EPI4605078	EPI4605071	EPI4605077	EPI4605073	EPI4605072
A/Yunnan-Jinghong/SWL178/2019	EPI_ISL_20111748	EPI4605067	EPI4605068	EPI4605066	EPI4605070	EPI4605063	EPI4605069	EPI4605065	EPI4605064
A/Yunnan-Linxiang/SWL1726/2019	EPI_ISL_20111747	EPI4605059	EPI4605060	EPI4605058	EPI4605062	EPI4605055	EPI4605061	EPI4605057	EPI4605056
A/Yunnan-Yuanjiang/SWL37/2019	EPI_ISL_20111746	EPI4605051	EPI4605052	EPI4605050	EPI4605054	EPI4605047	EPI4605053	EPI4605049	EPI4605048
A/Yunnan-Yuanjiang/SWL35/2019	EPI_ISL_20111745	EPI4605043	EPI4605044	EPI4605042	EPI4605046	EPI4605039	EPI4605045	EPI4605041	EPI4605040
A/Yunnan-Hongta/SWL159/2019	EPI_ISL_20111744	EPI4605035	EPI4605036	EPI4605034	EPI4605038	EPI4605031	EPI4605037	EPI4605033	EPI4605032
A/Yunnan-Linxiang/SWL11094/2019	EPI_ISL_20111743	EPI4605027	EPI4605028	EPI4605026	EPI4605030	EPI4605023	EPI4605029	EPI4605025	EPI4605024
A/Yunnan-Wenshan/SWL133/2019	EPI_ISL_20111742	EPI4605019	EPI4605020	EPI4605018	EPI4605022	EPI4605015	EPI4605021	EPI4605017	EPI4605016
A/Yunnan-Xishan/SWL127/2019	EPI_ISL_20111741	EPI4605011	EPI4605012	EPI4605010	EPI4605014	EPI4605007	EPI4605013	EPI4605009	EPI4605008
A/Yunnan-Linxiang/SWL1754/2019	EPI_ISL_20111739	EPI4605003	EPI4605004	EPI4605002	EPI4605006	EPI4604999	EPI4605005	EPI4605001	EPI4605000
A/Yunnan-Xishan/SWL129/2019	EPI_ISL_20111738	EPI4604995	EPI4604996	EPI4604994	EPI4604998	EPI4604991	EPI4604997	EPI4604993	EPI4604992
A/Yunnan-Qilin/SWL148/2019	EPI_ISL_20111737	EPI4604987	EPI4604988	EPI4604986	EPI4604990	EPI4604983	EPI4604989	EPI4604985	EPI4604984
A/Yunnan-Xianggelila/SWL11063/2019	EPI_ISL_20111736	EPI4604979	EPI4604980	EPI4604978	EPI4604982	EPI4604975	EPI4604981	EPI4604977	EPI4604976
A/Yunnan-Linxiang/SWL1657/2019	EPI_ISL_20111735	EPI4604971	EPI4604972	EPI4604970	EPI4604974	EPI4604967	EPI4604973	EPI4604969	EPI4604968
A/Yunnan-Fugong/SWL34/2019	EPI_ISL_20111734	EPI4604963	EPI4604964	EPI4604962	EPI4604966	EPI4604959	EPI4604965	EPI4604961	EPI4604960
A/Yunnan-Mangshi/SWL1798/2019	EPI_ISL_20111677	EPI4604900	EPI4604901	EPI4604899	EPI4604903	EPI4604896	EPI4604902	EPI4604898	EPI4604897
A/Yunnan-Jinghong/SWL157/2019	EPI_ISL_20111676	EPI4604892	EPI4604893	EPI4604891	EPI4604895	EPI4604888	EPI4604894	EPI4604890	EPI4604889
A/Yunnan-Wuhua/SWL1149/2019	EPI_ISL_20111675	EPI4604884	EPI4604885	EPI4604883	EPI4604887	EPI4604880	EPI4604886	EPI4604882	EPI4604881
A/Yunnan-Lushui/SWL1393/2019	EPI_ISL_20111674	EPI4604876	EPI4604877	EPI4604875	EPI4604879	EPI4604872	EPI4604878	EPI4604874	EPI4604873
A/Yunnan-Jinghong/SWL1231/2019	EPI_ISL_20111673	EPI4604868	EPI4604869	EPI4604867	EPI4604871	EPI4604864	EPI4604870	EPI4604866	EPI4604865
A/Yunnan-Wuhua/SWL110/2019	EPI_ISL_20111672	EPI4604860	EPI4604861	EPI4604859	EPI4604863	EPI4604856	EPI4604862	EPI4604858	EPI4604857
A/Yunnan-Wuhua/SWL18/2019	EPI_ISL_20111671	EPI4604852	EPI4604853	EPI4604851	EPI4604855	EPI4604848	EPI4604854	EPI4604850	EPI4604849
A/Yunnan-Hongta/SWL1735/2019	EPI_ISL_20111670	EPI4604844	EPI4604845	EPI4604843	EPI4604847	EPI4604840	EPI4604846	EPI4604842	EPI4604841
A/Yunnan-Anning/SWL1183/2019	EPI_ISL_20111669	EPI4604836	EPI4604837	EPI4604835	EPI4604839	EPI4604832	EPI4604838	EPI4604834	EPI4604833
A/Yunnan-Mengzi/SWL1160/2019	EPI_ISL_20111668	EPI4604828	EPI4604829	EPI4604827	EPI4604831	EPI4604824	EPI4604830	EPI4604826	EPI4604825

A/Yunnan-Xishan/SWL12/2019	EPI_ISL_20111667	EPI4604820	EPI4604821	EPI4604819	EPI4604823	EPI4604816	EPI4604822	EPI4604818	EPI4604817
A/Yunnan-Mengzi/SWL1236/2019	EPI_ISL_20111666	EPI4604812	EPI4604813	EPI4604811	EPI4604815	EPI4604808	EPI4604814	EPI4604810	EPI4604809
A/Yunnan-Wenshan/SWL1531/2019	EPI_ISL_20111665	EPI4604804	EPI4604805	EPI4604803	EPI4604807	EPI4604800	EPI4604806	EPI4604802	EPI4604801
A/Yunnan-Chuxiong/SWL11034/2019	EPI_ISL_20111664	EPI4604796	EPI4604797	EPI4604795	EPI4604799	EPI4604792	EPI4604798	EPI4604794	EPI4604793
A/Yunnan-Chuxiong/SWL156/2018	EPI_ISL_20111663	EPI4604788	EPI4604789	EPI4604787	EPI4604791	EPI4604784	EPI4604790	EPI4604786	EPI4604785
A/Yunnan-Yingjiang/SWL323/2018	EPI_ISL_20111662	EPI4604780	EPI4604781	EPI4604779	EPI4604783	EPI4604776	EPI4604782	EPI4604778	EPI4604777
A/Yunnan-Xianggelila/SWL1946/2018	EPI_ISL_20111661	EPI4604772	EPI4604773	EPI4604771	EPI4604775	EPI4604768	EPI4604774	EPI4604770	EPI4604769
A/Yunnan-Lushui/SWL1577/2018	EPI_ISL_20111660	EPI4604764	EPI4604765	EPI4604763	EPI4604767	EPI4604760	EPI4604766	EPI4604762	EPI4604761
A/Yunnan-Chuxiong/SWL1201/2018	EPI_ISL_20111659	EPI4604756	EPI4604757	EPI4604755	EPI4604759	EPI4604752	EPI4604758	EPI4604754	EPI4604753
A/Yunnan-Chuxiong/SWL1784/2018	EPI_ISL_20111658	EPI4604748	EPI4604749	EPI4604747	EPI4604751	EPI4604744	EPI4604750	EPI4604746	EPI4604745
A/Yunnan-Mengzi/SWL11027/2018	EPI_ISL_20111657	EPI4604740	EPI4604741	EPI4604739	EPI4604743	EPI4604736	EPI4604742	EPI4604738	EPI4604737
A/Yunnan-Chuxiong/SWL1829/2018	EPI_ISL_20111656	EPI4604732	EPI4604733	EPI4604731	EPI4604735	EPI4604728	EPI4604734	EPI4604730	EPI4604729
A/Yunnan-Hongta/SWL11542/2018	EPI_ISL_20111655	EPI4604724	EPI4604725	EPI4604723	EPI4604727	EPI4604720	EPI4604726	EPI4604722	EPI4604721
A/Yunnan-Mengzi/SWL11018/2018	EPI_ISL_20111654	EPI4604716	EPI4604717	EPI4604715	EPI4604719	EPI4604712	EPI4604718	EPI4604714	EPI4604713
A/Yunnan-Mengzi/SWL1990/2018	EPI_ISL_20111653	EPI4604708	EPI4604709	EPI4604707	EPI4604711	EPI4604704	EPI4604710	EPI4604706	EPI4604705
A/Yunnan-Lushui/SWL1836/2018	EPI_ISL_20111652	EPI4604700	EPI4604701	EPI4604699	EPI4604703	EPI4604696	EPI4604702	EPI4604698	EPI4604697
A/Yunnan-Hongta/SWL11514/2018	EPI_ISL_20111651	EPI4604692	EPI4604693	EPI4604691	EPI4604695	EPI4604688	EPI4604694	EPI4604690	EPI4604689
A/Yunnan-Hongta/SWL11533/2018	EPI_ISL_20111650	EPI4604684	EPI4604685	EPI4604683	EPI4604687	EPI4604680	EPI4604686	EPI4604682	EPI4604681
A/Yunnan-Hongta/SWL11530/2018	EPI_ISL_20111649	EPI4604676	EPI4604677	EPI4604675	EPI4604679	EPI4604672	EPI4604678	EPI4604674	EPI4604673
A/Yunnan-Hongta/SWL11517/2018	EPI_ISL_20111648	EPI4604668	EPI4604669	EPI4604667	EPI4604671	EPI4604664	EPI4604670	EPI4604666	EPI4604665
A/Yunnan-Hongta/SWL11523/2018	EPI_ISL_20111647	EPI4604660	EPI4604661	EPI4604659	EPI4604663	EPI4604656	EPI4604662	EPI4604658	EPI4604657
A/Yunnan-Hongta/SWL11501/2018	EPI_ISL_20111646	EPI4604652	EPI4604653	EPI4604651	EPI4604655	EPI4604648	EPI4604654	EPI4604650	EPI4604649
A/Yunnan-Dali/SWL1142/2018	EPI_ISL_20111645	EPI4604644	EPI4604645	EPI4604643	EPI4604647	EPI4604640	EPI4604646	EPI4604642	EPI4604641
A/Yunnan-Mangshi/SWL310/2018	EPI_ISL_20111644	EPI4604636	EPI4604637	EPI4604635	EPI4604639	EPI4604632	EPI4604638	EPI4604634	EPI4604633
A/Yunnan-Mangshi/SWL312/2018	EPI_ISL_20111643	EPI4604628	EPI4604629	EPI4604627	EPI4604631	EPI4604624	EPI4604630	EPI4604626	EPI4604625
A/Yunnan-Ruili/SWL3162/2018	EPI_ISL_20111642	EPI4604620	EPI4604621	EPI4604619	EPI4604623	EPI4604616	EPI4604622	EPI4604618	EPI4604617
A/Yunnan-Ruili/SWL3170/2018	EPI_ISL_20111641	EPI4604612	EPI4604613	EPI4604611	EPI4604615	EPI4604608	EPI4604614	EPI4604610	EPI4604609
A/Yunnan-Ruili/SWL359/2018	EPI_ISL_20111640	EPI4604604	EPI4604605	EPI4604603	EPI4604607	EPI4604600	EPI4604606	EPI4604602	EPI4604601
A/Yunnan-Ruili/SWL3138/2018	EPI_ISL_20111639	EPI4604596	EPI4604597	EPI4604595	EPI4604599	EPI4604592	EPI4604598	EPI4604594	EPI4604593
A/Yunnan-Ruili/SWL391/2018	EPI_ISL_20111638	EPI4604588	EPI4604589	EPI4604587	EPI4604591	EPI4604584	EPI4604590	EPI4604586	EPI4604585
A/Yunnan-Longyang/SWL11367/2018	EPI_ISL_20111637	EPI4604580	EPI4604581	EPI4604579	EPI4604583	EPI4604576	EPI4604582	EPI4604578	EPI4604577
A/Yunnan-Mangshi/SWL1636/2018	EPI_ISL_20111636	EPI4604572	EPI4604573	EPI4604571	EPI4604575	EPI4604568	EPI4604574	EPI4604570	EPI4604569
A/Yunnan-Chuxiong/SWL1211/2018	EPI_ISL_20111635	EPI4604564	EPI4604565	EPI4604563	EPI4604567	EPI4604560	EPI4604566	EPI4604562	EPI4604561
A/Yunnan-Chuxiong/SWL174/2018	EPI_ISL_20111634	EPI4604556	EPI4604557	EPI4604555	EPI4604559	EPI4604552	EPI4604558	EPI4604554	EPI4604553
A/Yunnan-Chuxiong/SWL133/2018	EPI_ISL_20111633	EPI4604548	EPI4604549	EPI4604547	EPI4604551	EPI4604544	EPI4604550	EPI4604546	EPI4604545
A/Yunnan-Chuxiong/SWL1170/2018	EPI_ISL_20111632	EPI4604540	EPI4604541	EPI4604539	EPI4604543	EPI4604536	EPI4604542	EPI4604538	EPI4604537
A/Yunnan-Lushui/SWL1606/2018	EPI_ISL_20111631	EPI4604532	EPI4604533	EPI4604531	EPI4604535	EPI4604528	EPI4604534	EPI4604530	EPI4604529
A/Yunnan-Lushui/SWL1580/2018	EPI_ISL_20111630	EPI4604524	EPI4604525	EPI4604523	EPI4604527	EPI4604520	EPI4604526	EPI4604522	EPI4604521
A/Yunnan-Lushui/SWL1614/2018	EPI_ISL_20111629	EPI4604516	EPI4604517	EPI4604515	EPI4604519	EPI4604512	EPI4604518	EPI4604514	EPI4604513
A/Yunnan-Mengzi/SWL181/2018	EPI_ISL_20111628	EPI4604508	EPI4604509	EPI4604507	EPI4604511	EPI4604504	EPI4604510	EPI4604506	EPI4604505
A/Yunnan-Jinping/SWL32/2018	EPI_ISL_20111627	EPI4604500	EPI4604501	EPI4604499	EPI4604503	EPI4604496	EPI4604502	EPI4604498	EPI4604497
A/Yunnan-Qilin/SWL1201/2018	EPI_ISL_20111626	EPI4604492	EPI4604493	EPI4604491	EPI4604495	EPI4604488	EPI4604494	EPI4604490	EPI4604489
A/Yunnan-Lushui/SWL1895/2018	EPI_ISL_20111625	EPI4604484	EPI4604485	EPI4604483	EPI4604487	EPI4604480	EPI4604486	EPI4604482	EPI4604481
A/Yunnan-Lushui/SWL1877/2018	EPI_ISL_20111624	EPI4604476	EPI4604477	EPI4604475	EPI4604479	EPI4604472	EPI4604478	EPI4604474	EPI4604473

A/Yunnan-Lushui/SWL1593/2018	EPI_ISL_20111623	EPI4604468	EPI4604469	EPI4604467	EPI4604471	EPI4604464	EPI4604470	EPI4604466	EPI4604465
A/Yunnan-Lushui/SWL1615/2018	EPI_ISL_20111622	EPI4604460	EPI4604461	EPI4604459	EPI4604463	EPI4604456	EPI4604462	EPI4604458	EPI4604457
A/Yunnan-Jingdong/SWL512/2018	EPI_ISL_20111621	EPI4604452	EPI4604453	EPI4604451	EPI4604455	EPI4604448	EPI4604454	EPI4604450	EPI4604449
A/Yunnan-Dali/SWL1165/2018	EPI_ISL_20111620	EPI4604444	EPI4604445	EPI4604443	EPI4604447	EPI4604440	EPI4604446	EPI4604442	EPI4604441
A/Yunnan-Chuxiong/SWL122/2018	EPI_ISL_20111619	EPI4604436	EPI4604437	EPI4604435	EPI4604439	EPI4604432	EPI4604438	EPI4604434	EPI4604433
A/Yunnan-Jinghong/SWL1955/2018	EPI_ISL_20111618	EPI4604428	EPI4604429	EPI4604427	EPI4604431	EPI4604424	EPI4604430	EPI4604426	EPI4604425
A/Yunnan-Dali/SWL1907/2018	EPI_ISL_20111617	EPI4604420	EPI4604421	EPI4604419	EPI4604423	EPI4604416	EPI4604422	EPI4604418	EPI4604417
A/Yunnan-Chuxiong/SWL1131/2018	EPI_ISL_20111616	EPI4604412	EPI4604413	EPI4604411	EPI4604415	EPI4604408	EPI4604414	EPI4604410	EPI4604409
A/Yunnan-Dayao/SWL33/2018	EPI_ISL_20111615	EPI4604404	EPI4604405	EPI4604403	EPI4604407	EPI4604400	EPI4604406	EPI4604402	EPI4604401
A/Yunnan-Chuxiong/SWL169/2018	EPI_ISL_20111614	EPI4604396	EPI4604397	EPI4604395	EPI4604399	EPI4604392	EPI4604398	EPI4604394	EPI4604393
A/Yunnan-Chuxiong/SWL1128/2018	EPI_ISL_20111613	EPI4604388	EPI4604389	EPI4604387	EPI4604391	EPI4604384	EPI4604390	EPI4604386	EPI4604385
A/Yunnan-Xishan/SWL1203/2018	EPI_ISL_20111612	EPI4604380	EPI4604381	EPI4604379	EPI4604383	EPI4604376	EPI4604382	EPI4604378	EPI4604377
A/Yunnan-Chuxiong/SWL62/2018	EPI_ISL_20111611	EPI4604372	EPI4604373	EPI4604371	EPI4604375	EPI4604368	EPI4604374	EPI4604370	EPI4604369
A/Yunnan-Hongta/SWL1812/2018	EPI_ISL_20111610	EPI4604364	EPI4604365	EPI4604363	EPI4604367	EPI4604360	EPI4604366	EPI4604362	EPI4604361
A/Yunnan-Chuxiong/SWL1140/2018	EPI_ISL_20111609	EPI4604356	EPI4604357	EPI4604355	EPI4604359	EPI4604352	EPI4604358	EPI4604354	EPI4604353
A/Yunnan-Zhaoyang/SWL187/2018	EPI_ISL_20111608	EPI4604348	EPI4604349	EPI4604347	EPI4604351	EPI4604344	EPI4604350	EPI4604346	EPI4604345
A/Yunnan-Gucheng/SWL1882/2018	EPI_ISL_20111607	EPI4604340	EPI4604341	EPI4604339	EPI4604343	EPI4604336	EPI4604342	EPI4604338	EPI4604337
A/Yunnan-Mangshi/SWL1595/2018	EPI_ISL_20111606	EPI4604332	EPI4604333	EPI4604331	EPI4604335	EPI4604328	EPI4604334	EPI4604330	EPI4604329
A/Yunnan-Gucheng/SWL1894/2018	EPI_ISL_20111605	EPI4604324	EPI4604325	EPI4604323	EPI4604327	EPI4604320	EPI4604326	EPI4604322	EPI4604321
A/Yunnan-Gucheng/SWL1924/2018	EPI_ISL_20111604	EPI4604316	EPI4604317	EPI4604315	EPI4604319	EPI4604312	EPI4604318	EPI4604314	EPI4604313
A/Yunnan-Gucheng/SWL1876/2018	EPI_ISL_20111603	EPI4604308	EPI4604309	EPI4604307	EPI4604311	EPI4604304	EPI4604310	EPI4604306	EPI4604305
A/Yunnan-Gucheng/SWL1856/2018	EPI_ISL_20111602	EPI4604300	EPI4604301	EPI4604299	EPI4604303	EPI4604296	EPI4604302	EPI4604298	EPI4604297
A/Yunnan-Dali/SWL11088/2018	EPI_ISL_20111601	EPI4604292	EPI4604293	EPI4604291	EPI4604295	EPI4604288	EPI4604294	EPI4604290	EPI4604289
A/Yunnan-Lushui/SWL1871/2018	EPI_ISL_20111600	EPI4604284	EPI4604285	EPI4604283	EPI4604287	EPI4604280	EPI4604286	EPI4604282	EPI4604281
A/Yunnan-Lushui/SWL1597/2018	EPI_ISL_20111599	EPI4604276	EPI4604277	EPI4604275	EPI4604279	EPI4604272	EPI4604278	EPI4604274	EPI4604273
A/Yunnan-Zhaoyang/SWL1448/2018	EPI_ISL_20111598	EPI4604268	EPI4604269	EPI4604267	EPI4604271	EPI4604264	EPI4604270	EPI4604266	EPI4604265
A/Yunnan-Wuhua/SWL1226/2018	EPI_ISL_20111597	EPI4604260	EPI4604261	EPI4604259	EPI4604263	EPI4604256	EPI4604262	EPI4604258	EPI4604257
A/Yunnan-Lushui/SWL1854/2018	EPI_ISL_20111596	EPI4604252	EPI4604253	EPI4604251	EPI4604255	EPI4604248	EPI4604254	EPI4604250	EPI4604249
A/Yunnan-Lushui/SWL31/2018	EPI_ISL_20111595	EPI4604244	EPI4604245	EPI4604243	EPI4604247	EPI4604240	EPI4604246	EPI4604242	EPI4604241
A/Yunnan-Lushui/SWL1784/2018	EPI_ISL_20111594	EPI4604236	EPI4604237	EPI4604235	EPI4604239	EPI4604232	EPI4604238	EPI4604234	EPI4604233
A/Yunnan-Lushui/SWL1833/2018	EPI_ISL_20111593	EPI4604228	EPI4604229	EPI4604227	EPI4604231	EPI4604224	EPI4604230	EPI4604226	EPI4604225
A/Yunnan-Lushui/SWL1825/2018	EPI_ISL_20111592	EPI4604220	EPI4604221	EPI4604219	EPI4604223	EPI4604216	EPI4604222	EPI4604218	EPI4604217
A/Yunnan-Lushui/SWL1787/2018	EPI_ISL_20111591	EPI4604212	EPI4604213	EPI4604211	EPI4604215	EPI4604208	EPI4604214	EPI4604210	EPI4604209
A/Yunnan-Lushui/SWL39/2018	EPI_ISL_20111590	EPI4604204	EPI4604205	EPI4604203	EPI4604207	EPI4604200	EPI4604206	EPI4604202	EPI4604201
A/Yunnan-Guandu/SWL1815/2018	EPI_ISL_20111589	EPI4604196	EPI4604197	EPI4604195	EPI4604199	EPI4604192	EPI4604198	EPI4604194	EPI4604193
A/Yunnan-Wenshan/SWL167/2018	EPI_ISL_20111588	EPI4604188	EPI4604189	EPI4604187	EPI4604191	EPI4604184	EPI4604190	EPI4604186	EPI4604185
A/Yunnan-Gucheng/SWL1938/2018	EPI_ISL_20111587	EPI4604180	EPI4604181	EPI4604179	EPI4604183	EPI4604176	EPI4604182	EPI4604178	EPI4604177
A/Yunnan-Chuxiong/SWL128/2018	EPI_ISL_20111586	EPI4604172	EPI4604173	EPI4604171	EPI4604175	EPI4604168	EPI4604174	EPI4604170	EPI4604169
A/Yunnan-Mangshi/SWL1578/2018	EPI_ISL_20111585	EPI4604164	EPI4604165	EPI4604163	EPI4604167	EPI4604160	EPI4604166	EPI4604162	EPI4604161
A/Yunnan-Mangshi/SWL1609/2018	EPI_ISL_20111584	EPI4604156	EPI4604157	EPI4604155	EPI4604159	EPI4604152	EPI4604158	EPI4604154	EPI4604153
A/Yunnan-Mangshi/SWL1586/2018	EPI_ISL_20111583	EPI4604148	EPI4604149	EPI4604147	EPI4604151	EPI4604144	EPI4604150	EPI4604146	EPI4604145
A/Yunnan-Chuxiong/SWL136/2018	EPI_ISL_20111582	EPI4604140	EPI4604141	EPI4604139	EPI4604143	EPI4604136	EPI4604142	EPI4604138	EPI4604137
A/Yunnan-Chuxiong/SWL161/2018	EPI_ISL_20111581	EPI4604132	EPI4604133	EPI4604131	EPI4604135	EPI4604128	EPI4604134	EPI4604130	EPI4604129
A/Yunnan-Chuxiong/SWL145/2018	EPI_ISL_20111580	EPI4604124	EPI4604125	EPI4604123	EPI4604127	EPI4604120	EPI4604126	EPI4604122	EPI4604121

A/Yunnan-Chuxiong/SWL1163/2018	EPI_ISL_20111579	EPI4604116	EPI4604117	EPI4604115	EPI4604119	EPI4604112	EPI4604118	EPI4604114	EPI4604113
A/California/7/2009	EPI_ISL_391380	EPI1593566	EPI1593567	EPI1593565	EPI1593569	EPI1593562	EPI1593568	EPI1593564	EPI1593563
A/Michigan/45/2015	EPI_ISL_199532	EPI662591	EPI662592	EPI662590	EPI662594	EPI662587	EPI662593	EPI662589	EPI662588
A/Brisbane/02/2018	EPI_ISL_330190	EPI1312563	EPI1312564	EPI1312562	EPI1312566	EPI1312559	EPI1312565	EPI1312561	EPI1312560
A/Hawaii/70/2019	EPI_ISL_400916	EPI1642228	EPI1642229	EPI1642227	EPI1642231	EPI1642224	EPI1642230	EPI1642226	EPI1642225
A/Wisconsin/588/2019	EPI_ISL_404460	EPI1661228	EPI1661229	EPI1661227	EPI1661231	EPI1661224	EPI1661230	EPI1661226	EPI1661225
A/Wisconsin/67/2022	EPI_ISL_15928538	EPI2224785	EPI2224786	EPI2224784	EPI2224788	EPI2224781	EPI2224787	EPI2224783	EPI2224782
A/KANAGAWA/IC1832/2019	EPI_ISL_354781	EPI1436157	EPI1436158	EPI1436156	EPI1436160	EPI1436153	EPI1436159	EPI1436155	EPI1436154
A/Singapore/GP1574/2017	EPI_ISL_281331	EPI1076780	EPI1076781	EPI1076779	EPI1076783	EPI1076776	EPI1076782	EPI1076778	EPI1076777
A/Bourgogne/2191/2018	EPI_ISL_330738	EPI1315835	EPI1315836	EPI1315834	EPI1315838	EPI1315831	EPI1315837	EPI1315833	EPI1315832
A/Iowa/77/2018	EPI_ISL_336671	EPI1349817	EPI1349818	EPI1349816	EPI1349820	EPI1349813	EPI1349819	EPI1349815	EPI1349814
A/Moscow/171/2019	EPI_ISL_377976	EPI1547662	EPI1547663	EPI1547661	EPI1547665	EPI1547658	EPI1547664	EPI1547660	EPI1547659
A/Hong Kong/DRV086/2023	EPI_ISL_19232716	EPI3413953	EPI3413951	EPI3413948	EPI3413949	EPI3413950	EPI3413947	EPI3413954	EPI3413952
A/Bhutan/211/2019	EPI_ISL_368176	EPI1507963	EPI1507967	EPI1507962	EPI1507974	EPI1507959	EPI1507970	EPI1507961	EPI1507960
A/Laos/2591/2018	EPI_ISL_364136	EPI1488415	EPI1488416	EPI1488414	EPI1488418	EPI1488412	EPI1488417	EPI1488413	EPI1490622
A/South Africa/R08500/2018	EPI_ISL_18765111	EPI2915309	EPI2915307	EPI2915304	EPI2915305	EPI2915306	EPI2915303	EPI2915310	EPI2915308
A/Alaska/18/2017	EPI_ISL_273769	EPI1039632	EPI1039633	EPI1039631	EPI1039635	EPI1039628	EPI1039634	EPI1039630	EPI1039629
A/Anhui-Jinghu/SWL6116/2017	EPI_ISL_18760232	EPI2914277	EPI2914278	EPI2914276	EPI2914280	EPI2914273	EPI2914279	EPI2914275	EPI2914274
A/Anhui-Jinghu/SWL11057/2017	EPI_ISL_18760220	EPI2914181	EPI2914182	EPI2914180	EPI2914184	EPI2914177	EPI2914183	EPI2914179	EPI2914178
A/California/34/2019	EPI_ISL_342309	EPI1373004	EPI1373005	EPI1373003	EPI1373007	EPI1373000	EPI1373006	EPI1373002	EPI1373001
A/Eskilstuna/6/2018	EPI_ISL_336145	EPI1346086	EPI1346085	EPI1346083	EPI1346090	EPI1346084	EPI1346089	EPI1346088	EPI1346087
A/HIROSHIMA/90/2023	EPI_ISL_18139324	EPI2716000	EPI2716001	EPI2715999	EPI2716003	EPI2715996	EPI2716002	EPI2715998	EPI2715997
A/Hong Kong/CUHK1049/2019	EPI_ISL_18623991	EPI2838450	EPI2838451	EPI2838449	EPI2838453	EPI2838446	EPI2838452	EPI2838448	EPI2838447
A/Jinan/JN_05/2019	EPI_ISL_20299630	EPI4921206	EPI4921204	EPI4921201	EPI4921202	EPI4921203	EPI4921200	EPI4921207	EPI4921205
A/Kazakhstan/18/2019	EPI_ISL_366029	EPI1497777	EPI1497778	EPI1497776	EPI1497780	EPI1497773	EPI1497779	EPI1497775	EPI1497774
A/Kyiv/29/2020	EPI_ISL_428216	EPI1731613	EPI1731614	EPI1731612	EPI1731616	EPI1731609	EPI1731615	EPI1731611	EPI1731610
A/Laos/3339/2018	EPI_ISL_36815	EPI1507774	EPI1507775	EPI1507773	EPI1507777	EPI1507770	EPI1507776	EPI1507772	EPI1507771
A/Laos/3389/2018	EPI_ISL_368155	EPI1507798	EPI1507799	EPI1507797	EPI1507801	EPI1507794	EPI1507800	EPI1507796	EPI1507795
A/Laos/3855/2016	EPI_ISL_269945	EPI1025086	EPI1025087	EPI1025085	EPI1025089	EPI1025082	EPI1025088	EPI1025084	EPI1025083
A/Michigan/13/2020	EPI_ISL_2271169	EPI1866263	EPI1866264	EPI1866262	EPI1866266	EPI1866259	EPI1866265	EPI1866261	EPI1866260
A/New Zealand/18CF0074/2018	EPI_ISL_20171275	EPI4690568	EPI4690566	EPI4690563	EPI4690564	EPI4690565	EPI4690562	EPI4690569	EPI4690567
A/New Zealand/18CF0739/2018	EPI_ISL_20171226	EPI4690185	EPI4690183	EPI4690180	EPI4690181	EPI4690182	EPI4690179	EPI4690186	EPI4690184
A/Nigeria/3626/2018	EPI_ISL_353346	EPI1427107	EPI1427108	EPI1427106	EPI1427110	EPI1427103	EPI1427109	EPI1427105	EPI1427104
A/Oklahoma/28/2018	EPI_ISL_336667	EPI1349785	EPI1349786	EPI1349784	EPI1349788	EPI1349781	EPI1349787	EPI1349783	EPI1349782
A/Pakistan/122/2019	EPI_ISL_351836	EPI1420559	EPI1420560	EPI1420558	EPI1420562	EPI1420555	EPI1420561	EPI1420557	EPI1420556
A/Panama/M270103/2023	EPI_ISL_18021939	EPI2646049	EPI2646046	EPI2646039	EPI2646043	EPI2646045	EPI2646037	EPI2646051	EPI2646047
A/Pennsylvania/159/2020	EPI_ISL_2588613	EPI1876832	EPI1876833	EPI1876831	EPI1876835	EPI1876828	EPI1876834	EPI1876830	EPI1876829
A/Singapore/TT0676/2018	EPI_ISL_340460	EPI1394609	EPI1394610	EPI1394608	EPI1365920	EPI1394605	EPI1365919	EPI1394607	EPI1394606
A/Vietnam/Lisn214/2017	EPI_ISL_292683	EPI1147204	EPI1147205	EPI1147203	EPI1147207	EPI1147200	EPI1147206	EPI1147202	EPI1147201
A/Washington/S26338/2019	EPI_ISL_19607512	EPI3710550	EPI3710548	EPI3710545	EPI3710546	EPI3710547	EPI3710544	EPI3710551	EPI3710549
A/Washington/S26998/2020	EPI_ISL_19606002	EPI3700677	EPI3700675	EPI3700672	EPI3700673	EPI3700674	EPI3700671	EPI3700678	EPI3700676

Abbreviations: PB2, polymerase basic 2; PB1, polymerase basic 1; PA, polymerase acid; HA, hemagglutinin; NP, nucleocapsid protein; NA, neuraminidase; M, matrix protein; NS, nonstructural protein.
