# Supplementary material for: Genomic insights into influenza A(H1N1)pdm09 virus evolution in Yunnan province, China, during 2018–2023
Source: Virol J. 2026 Jul 3;23:222. doi: 10.1186/s12985-026-03238-7 (PMC13602790; doi:10.1186/s12985-026-03238-7)
Supplement: Supplementary file 2 [file 12985_2026_3238_MOESM2_ESM.pdf]

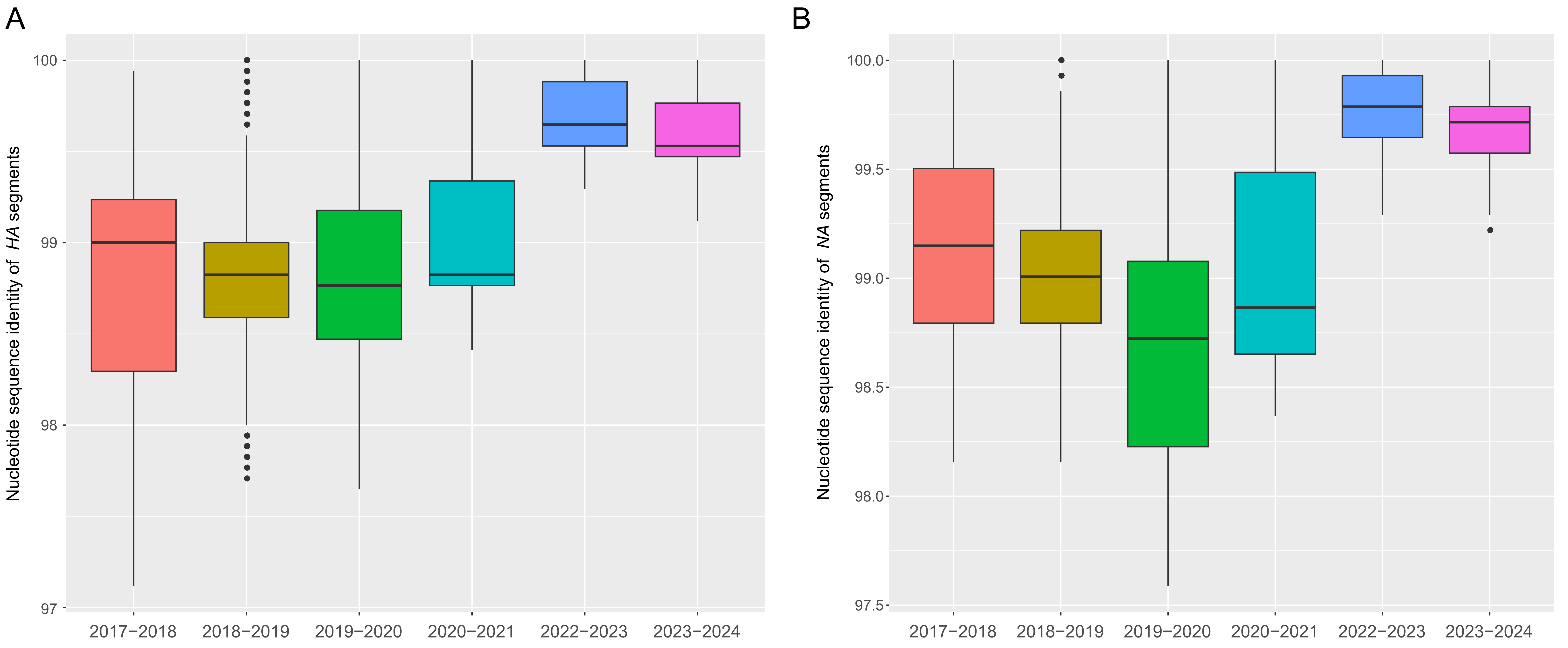

A
Nucleotide sequence identity of HA segments
100
99
98
97
2017−2018
2018−2019
2019−2020
2020−2021
2022−2023
2023−2024
B
Nucleotide sequence identity of NA segments
100.0
99.5
99.0
98.5
98.0
97.5
2017−2018
2018−2019
2019−2020
2020−2021
2022−2023
2023−2024
