# Supplementary material for: Genomic insights into influenza A(H1N1)pdm09 virus evolution in Yunnan province, China, during 2018–2023
Source: Virol J. 2026 Jul 3;23:222. doi: 10.1186/s12985-026-03238-7 (PMC13602790; doi:10.1186/s12985-026-03238-7)
Supplement: Supplementary file 3 [file 12985_2026_3238_MOESM3_ESM.pdf]

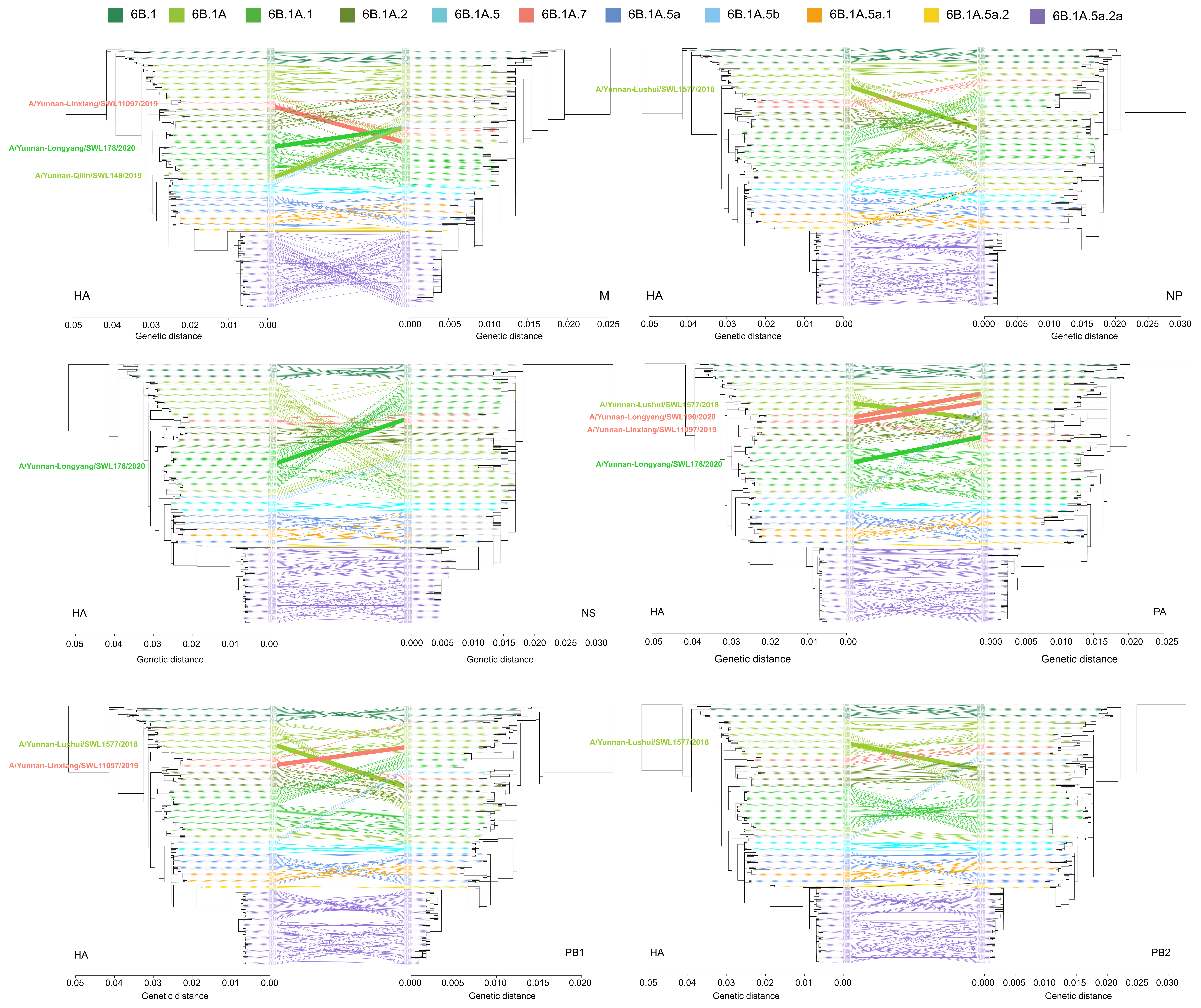
6B.1
6B.1A
6B.1A.1
6B.1A.2
6B.1A.5
6B.1A.7
6B.1A.5a
6B.1A.5b
6B.1A.5a.1
6B.1A.5a.2
6B.1A.5a.2a
A/Yunnan-Linxiang/SWL11097/2019
A/Yunnan-Longyang/SWL178/2020
A/Yunnan-Qilin/SWL148/2019
HA
M
0.05 0.04 0.03 0.02 0.01 0.00
Genetic distance
0.000 0.005 0.010 0.015 0.020 0.025
Genetic distance
A/Yunnan-Lushui/SWL1577/2018
HA
NP
0.05 0.04 0.03 0.02 0.01 0.00
Genetic distance
0.000 0.005 0.010 0.015 0.020 0.025 0.030
Genetic distance
A/Yunnan-Longyang/SWL178/2020
HA
NS
0.05 0.04 0.03 0.02 0.01 0.00
Genetic distance
0.000 0.005 0.010 0.015 0.020 0.025 0.030
Genetic distance
A/Yunnan-Lushui/SWL1577/2018
A/Yunnan-Longyang/SWL190/2020
A/Yunnan-Linxiang/SWL11097/2019
A/Yunnan-Longyang/SWL178/2020
HA
PA
0.05 0.04 0.03 0.02 0.01 0.00
Genetic distance
0.000 0.005 0.010 0.015 0.020 0.025
Genetic distance
A/Yunnan-Lushui/SWL1577/2018
A/Yunnan-Linxiang/SWL11097/2019
HA
PB1
0.05 0.04 0.03 0.02 0.01 0.00
Genetic distance
0.000 0.005 0.010 0.015 0.020
Genetic distance
A/Yunnan-Lushui/SWL1577/2018
HA
PB2
0.05 0.04 0.03 0.02 0.01 0.00
Genetic distance
0.000 0.005 0.010 0.015 0.020 0.025 0.030
Genetic distance
