# Supplementary material for: Genomic insights into influenza A(H1N1)pdm09 virus evolution in Yunnan province, China, during 2018–2023
Source: Virol J. 2026 Jul 3;23:222. doi: 10.1186/s12985-026-03238-7 (PMC13602790; doi:10.1186/s12985-026-03238-7)
Supplement: Supplementary file 5 [file 12985_2026_3238_MOESM5_ESM.pdf]

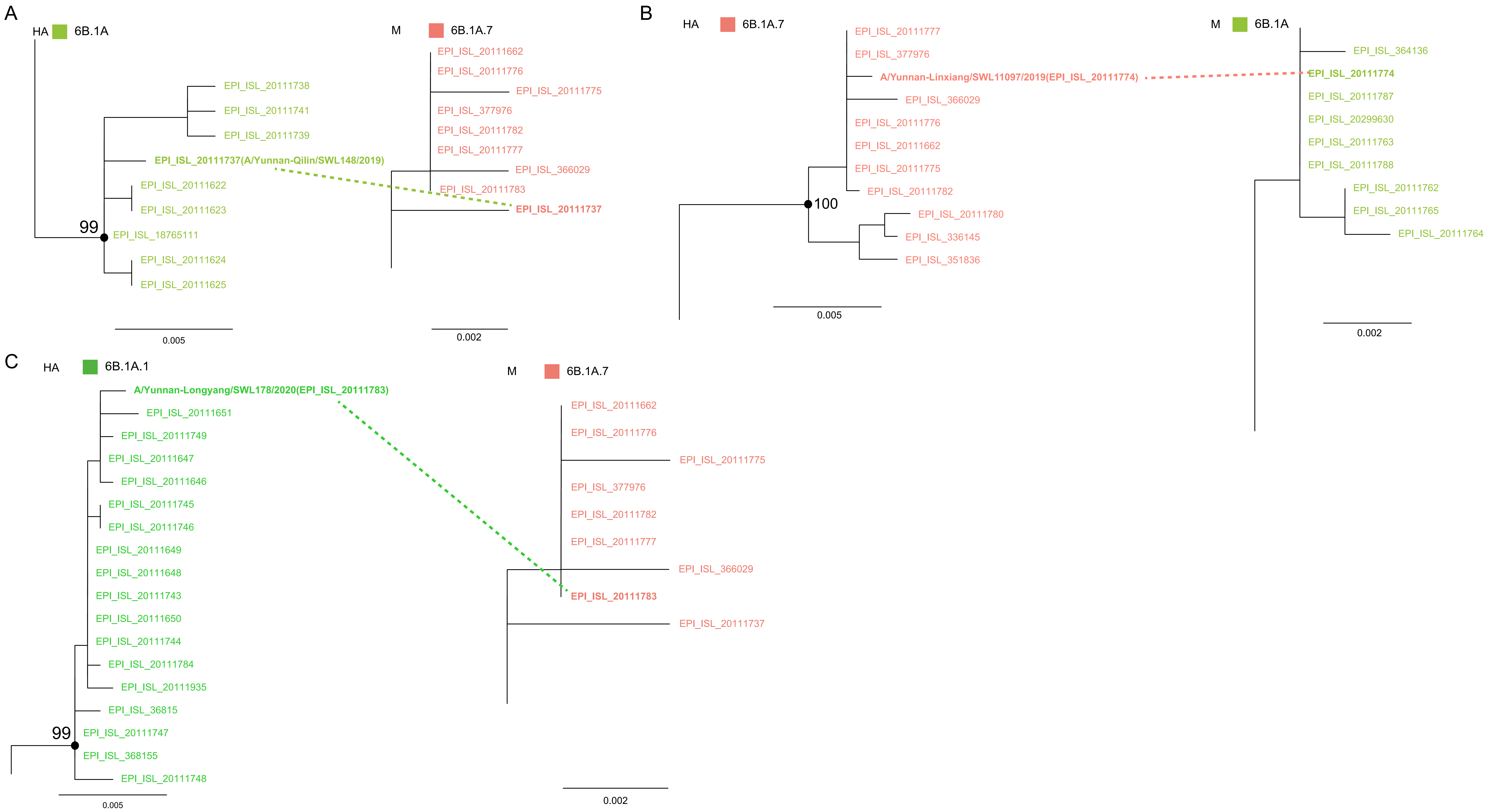

A
HA 6B.1A
EPI_ISL_20111738
EPI_ISL_20111741
EPI_ISL_20111739
EPI_ISL_20111737(A/Yunnan-Qilin/SWL148/2019)
EPI_ISL_20111622
EPI_ISL_20111623
99
EPI_ISL_18765111
EPI_ISL_20111624
EPI_ISL_20111625
0.005
M 6B.1A.7
EPI_ISL_20111662
EPI_ISL_20111776
EPI_ISL_20111775
EPI_ISL_377976
EPI_ISL_20111782
EPI_ISL_20111777
EPI_ISL_366029
EPI_ISL_20111783
EPI_ISL_20111737
0.002
B
HA 6B.1A.7
EPI_ISL_20111777
EPI_ISL_377976
A/Yunnan-Linxiang/SWL11097/2019(EPI_ISL_20111774)
EPI_ISL_366029
EPI_ISL_20111776
EPI_ISL_20111662
EPI_ISL_20111775
EPI_ISL_20111782
100
EPI_ISL_20111780
EPI_ISL_336145
EPI_ISL_351836
0.005
M 6B.1A
EPI_ISL_364136
EPI_ISL_20111774
EPI_ISL_20111787
EPI_ISL_20299630
EPI_ISL_20111763
EPI_ISL_20111788
EPI_ISL_20111762
EPI_ISL_20111765
EPI_ISL_20111764
0.002
C
HA 6B.1A.1
A/Yunnan-Longyang/SWL178/2020(EPI_ISL_20111783)
EPI_ISL_20111651
EPI_ISL_20111749
EPI_ISL_20111647
EPI_ISL_20111646
EPI_ISL_20111745
EPI_ISL_20111746
EPI_ISL_20111649
EPI_ISL_20111648
EPI_ISL_20111743
EPI_ISL_20111650
EPI_ISL_20111744
EPI_ISL_20111784
EPI_ISL_20111935
EPI_ISL_36815
99
EPI_ISL_20111747
EPI_ISL_368155
EPI_ISL_20111748
0.005
M 6B.1A.7
EPI_ISL_20111662
EPI_ISL_20111776
EPI_ISL_20111775
EPI_ISL_377976
EPI_ISL_20111782
EPI_ISL_20111777
EPI_ISL_366029
EPI_ISL_20111783
EPI_ISL_20111737
0.002
