# Supplementary material for: Genomic insights into influenza A(H1N1)pdm09 virus evolution in Yunnan province, China, during 2018–2023
Source: Virol J. 2026 Jul 3;23:222. doi: 10.1186/s12985-026-03238-7 (PMC13602790; doi:10.1186/s12985-026-03238-7)
Supplement: Supplementary file 6 [file 12985_2026_3238_MOESM6_ESM.pdf]

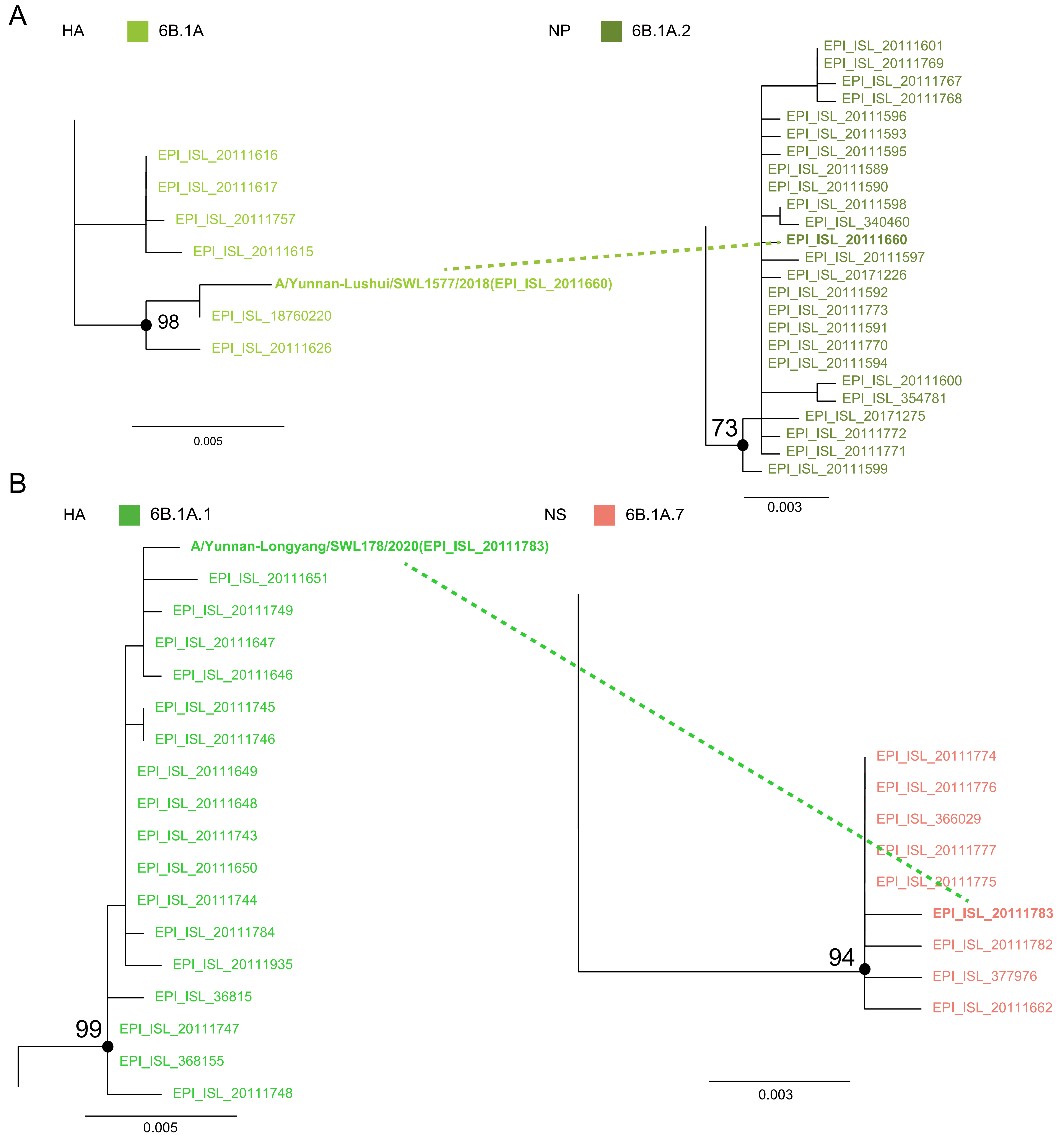

A
HA
6B.1A
NP
6B.1A.2
EPI_ISL_20111616
EPI_ISL_20111617
EPI_ISL_20111757
EPI_ISL_20111615
A/Yunnan-Lushui/SWL1577/2018(EPI_ISL_2011660)
EPI_ISL_18760220
98
EPI_ISL_20111626
0.005
EPI_ISL_20111601
EPI_ISL_20111769
EPI_ISL_20111767
EPI_ISL_20111768
EPI_ISL_20111596
EPI_ISL_20111593
EPI_ISL_20111595
EPI_ISL_20111589
EPI_ISL_20111590
EPI_ISL_20111598
EPI_ISL_340460
EPI_ISL_20111660
EPI_ISL_20111597
EPI_ISL_20171226
EPI_ISL_20111592
EPI_ISL_20111773
EPI_ISL_20111591
EPI_ISL_20111770
EPI_ISL_20111594
EPI_ISL_20111600
EPI_ISL_354781
EPI_ISL_20171275
73
EPI_ISL_20111772
EPI_ISL_20111771
EPI_ISL_20111599
0.003
B
HA
6B.1A.1
NS
6B.1A.7
A/Yunnan-Longyang/SWL178/2020(EPI_ISL_20111783)
EPI_ISL_20111651
EPI_ISL_20111749
EPI_ISL_20111647
EPI_ISL_20111646
EPI_ISL_20111745
EPI_ISL_20111746
EPI_ISL_20111649
EPI_ISL_20111648
EPI_ISL_20111743
EPI_ISL_20111650
EPI_ISL_20111744
EPI_ISL_20111784
EPI_ISL_20111935
EPI_ISL_36815
99
EPI_ISL_20111747
EPI_ISL_368155
EPI_ISL_20111748
0.005
EPI_ISL_20111774
EPI_ISL_20111776
EPI_ISL_366029
EPI_ISL_20111777
EPI_ISL_20111775
EPI_ISL_20111783
EPI_ISL_20111782
94
EPI_ISL_377976
EPI_ISL_20111662
0.003
